# Multiomics Analysis Reveals the Impact of Microbiota on Host Metabolism in Hepatic Steatosis

**DOI:** 10.1002/advs.202104373

**Published:** 2022-02-07

**Authors:** Mujdat Zeybel, Muhammad Arif, Xiangyu Li, Ozlem Altay, Hong Yang, Mengnan Shi, Murat Akyildiz, Burcin Saglam, Mehmet Gokhan Gonenli, Buket Yigit, Burge Ulukan, Dilek Ural, Saeed Shoaie, Hasan Turkez, Jens Nielsen, Cheng Zhang, Mathias Uhlén, Jan Borén, Adil Mardinoglu

**Affiliations:** ^1^ Department of Gastroenterology and Hepatology School of Medicine Koç University Istanbul 34010 Turkey; ^2^ NIHR Nottingham Biomedical Research Centre Nottingham University Hospitals NHS Trust & University of Nottingham Nottingham NG5 1PB UK; ^3^ Nottingham Digestive Diseases Centre School of Medicine University of Nottingham Nottingham NG7 2UH UK; ^4^ Science for Life Laboratory KTH – Royal Institute of Technology Stockholm SE‐17121 Sweden; ^5^ School of Medicine Koç University Istanbul 34010 Turkey; ^6^ Centre for Host‐Microbiome Interactions Faculty of Dentistry, Oral & Craniofacial Sciences King's College London London SE1 9RT UK; ^7^ Department of Medical Biology Faculty of Medicine Atatürk University Erzurum 25240 Turkey; ^8^ Department of Biology and Biological Engineering Chalmers University of Technology Gothenburg SE‐41296 Sweden; ^9^ Key Laboratory of Advanced Drug Preparation Technologies Ministry of Education School of Pharmaceutical Sciences Zhengzhou University Zhengzhou Henan Province 450001 China; ^10^ Department of Molecular and Clinical Medicine University of Gothenburg and Sahlgrenska University Hospital Gothenburg Gothenburg SE‐41345 Sweden; ^11^ Present address: Laboratory of Cardiovascular Physiology and Tissue Injury and Section on Fibrotic Disorders National Institute on Alcohol Abuse and Alcoholism, National Institutes of Health Rockville MD 20852 USA

**Keywords:** gut and oral metagenomics, metabolic dysfunction‐associated fatty liver disease, metabolomics, multiomics analysis, proteomics, systems biology, systems medicine

## Abstract

Metabolic dysfunction‐associated fatty liver disease (MAFLD) is a complex disease involving alterations in multiple biological processes regulated by the interactions between obesity, genetic background, and environmental factors including the microbiome. To decipher hepatic steatosis (HS) pathogenesis by excluding critical confounding factors including genetic variants and diabetes, 56 heterogenous MAFLD patients are characterized by generating multiomics data including oral and gut metagenomics as well as plasma metabolomics and inflammatory proteomics data. The dysbiosis in the oral and gut microbiome is explored and the host–microbiome interactions based on global metabolic and inflammatory processes are revealed. These multiomics data are integrated using the biological network and HS's key features are identified using multiomics data. HS is finally predicted using these key features and findings are validated in a follow‐up cohort, where 22 subjects with varying degree of HS are characterized.

## Graphical abstract

A comprehensive study to decipher hepatic steatosis (HS) by generating multiomics data including oral and gut metagenomics as well as plasma metabolomics and inflammatory proteomics data in a well‐characterized metabolic dysfunction‐associated fatty liver disease (MAFLD) cohort.

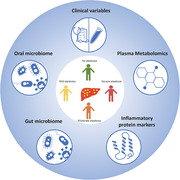

## 1 Introduction

Nonalcoholic fatty liver disease (NAFLD) is characterized by deposition of lipid droplets in the liver without significant alcohol consumption and secondary causes, which now has been defined as metabolic dysfunction‐associated fatty liver disease (MAFLD). MAFLD constitutes a wide range of the clinical spectrum, including hepatic steatosis (HS) and nonalcoholic steatohepatitis (NASH), which may ultimately lead to advanced fibrosis and cirrhosis. In parallel to ongoing epidemics of obesity, MAFLD incidence is increasing globally, affecting almost one‐fourth of the population.^[^ ^1^ ^]^ Even though MAFLD is becoming a leading etiology of chronic liver disease, therapeutic approaches for MAFLD are currently limited with lifestyle modifications encompassing dietary intervention and physical activity.^[^ ^2^ ^]^

Given that MAFLD is closely linked to metabolic syndrome, obesity, and type 2 diabetes (T2D), its pathogenesis is remarkably complex. Discoveries of *PNPLA3* and *TM6SF2* variants have highlighted the pathological processes leading to metabolic disturbances in HS. However, only 10–20% of MAFLD susceptibility could be attributed to known genetic variants.^[^ ^3^ ^]^ Exploring the connections between the hepatic and extra‐hepatic metabolic factors through the generation of the multiomics data may reveal the underlying molecular mechanisms associated with the disease's occurrence, discovering novel biomarkers and drug targets, and eventually provide further insight for the development of efficient treatment strategies. The dysbiosis and diversity in the oral and gut microbiome have been associated with MAFLD^[^ ^4^ ^]^ as well as metabolic syndrome,^[^ ^5^ ^]^ type 2 diabetes,^[^ ^6^ ^]^ and obesity.^[^ ^7^ ^]^ Studies indicated that microbiome composition changes are associated with advanced hepatic fibrosis^[^ ^8^ ^]^ and cirrhosis.^[^ ^9^ ^]^ Moreover, several studies integrated multiomics data across various environmental states through systems biology. These studies demonstrated that a multiomics approach is a powerful tool for understanding the dynamics of biological functions in liver diseases and other associated metabolic conditions.^[^ ^10^ ^]^

Here, we generated detailed clinical data and multiomics data (plasma metabolomics, plasma proteomics, oral microbiome, and gut microbiome) for 56 subjects to characterize the patients with different levels of hepatic steatosis. We also performed an integrated analysis and then deciphered the pathogenesis of early stage MAFLD. We collected saliva and feces samples for studying the dysbiosis in the oral and gut microbiome through the generation of shotgun metagenomics data and identified the key species involved in various stages of HS. Moreover, we performed plasma metabolomics and inflammatory proteomics analysis to explore the host–microbiome interactions. We studied the altered global metabolic and inflammatory processes, and its connections with the species' abundances in the oral and gut microbiome. We integrated this multiomics data using biological networks and identified the key features of HS. We finally predicted HS using these key features and validated our findings in a follow‐up cohort including 22 subjects with varying HS.

## 2 Results

### 2.1 The Associations between HS and Clinical and Physical Variables

**Figure 1:**
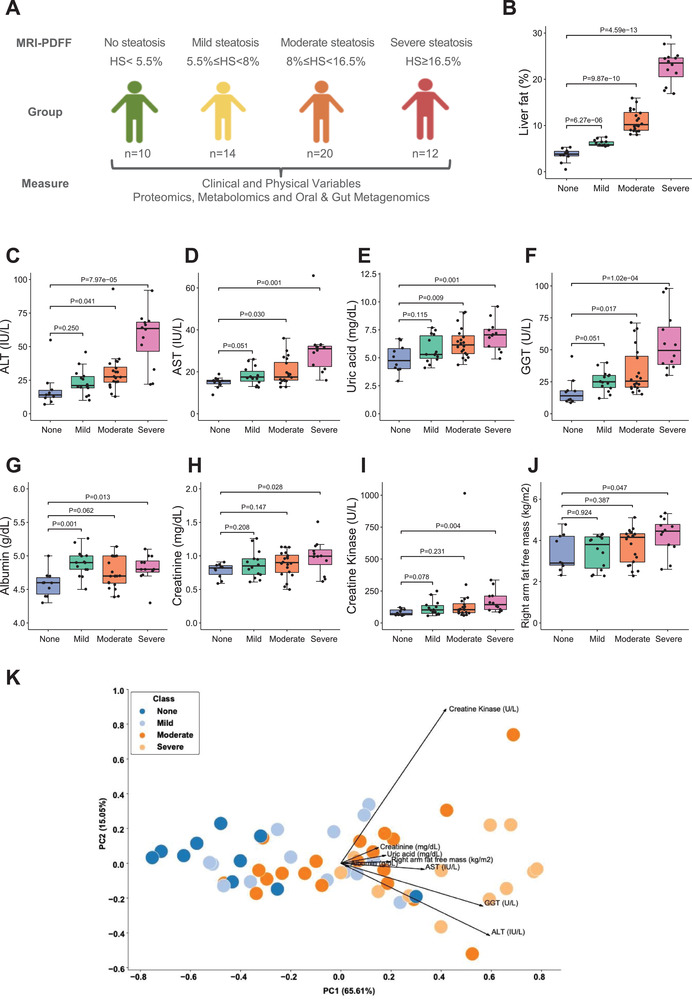
A) The design of the study. Subjects were stratified into four distinct groups based on the hepatic steatosis percentages measured using MRI‐PDFF. The sample sizes of none, mild, moderate, and severe steatosis groups are 10, 14, 20, and 12, respectively. B) The boxplot shows hepatic steatosis of subjects with none, mild, moderate, and severe steatosis. Student's *t*‐test was used for statistical analysis. C–J) Significantly different clinical parameters are presented in subjects with none, mild, moderate, and severe steatosis. Student's *t*‐test was used for statistical analysis. K) PCA of all subjects based on eight significantly different clinical and physical variables shows good separation.

We generated multiomics data for 56 overweight and obese subjects (body mass index (BMI) > 28.8 kg m^−2^) with a varying degree of HS. We excluded patients if they carry PNPLA3 I148M (homozygous for I148M). We determined HS by the proton density fat fraction, measured by magnetic resonance imaging (MRI‐PDFF). We classified the subjects into four different groups: i) 10 subjects with no steatosis (HS < 5.5%), ii) 14 subjects with mild steatosis (5.5% ≤ HS < 8%), ii) 20 subjects with moderate steatosis (8.0% ≤HS < 16.5%), and iv) 12 subjects with severe steatosis (HS ≥ 16.5%) (**Figure** **1**A,B). We carefully phenotyped these subjects by measuring clinical and physical variables (Dataset S1A–C, Supporting Information). We also collected saliva and feces samples to generate metagenomics data and plasma samples to generate metabolomics and inflammatory proteomics data. The subjects' demographic characteristics in each group are presented (Dataset S1C,D, Supporting Information).

To confirm our findings based on multiomics analysis of 56 subjects and avoid the genetic differences between the subjects, we re‐analyzed 22 of these patients after 2–3 months, generated clinical data, and measured the HS, denoted as follow‐up cohort. We observed that the degree of HS in each subject has changed since they have been recommended changes in their exercise and eating habits. The subjects' demographic characteristics in each group are presented (Dataset S2A–D, Supporting Information). These 22 subjects were classified based on HS as i) two subjects with no steatosis, ii) five subjects with mild steatosis, iii) eight subjects with moderate steatosis, and iv) seven subjects with severe steatosis. In the follow‐up cohort, we also generated oral (Dataset S2E, Supporting Information) and gut (Dataset S2F, Supporting Information) metagenomics, metabolomics (Dataset S2G, Supporting Information), and proteomics (Dataset S2H, Supporting Information) data using a similar methodology as in the finding dataset. We compared the difference between the clinical characteristics of the patients with HS degree from the overall and follow‐up cohorts. The result showed that there was no difference in clinical parameters except serum total bilirubin between no steatosis patients from the two cohorts (Student's *t*‐test, *p* < 0.05, Dataset S2I, Supporting Information).

We analyzed the differences in the clinical and physical variables between the groups with different HS degrees in the overall dataset (Dataset S1C, Supporting Information). We did not find any differences in the weight, BMI, waist circumference, homeostasis model assessment‐estimated insulin resistance score, glucose, insulin, and HbA1c levels between different steatosis groups. We found that the levels of alanine aminotransferase (ALT; Figure 1C), aspartate aminotransferase (AST; Figure 1D), uric acid (urate; Figure 1E), and gamma‐glutamyl transferase (GGT; Figure 1F) were significantly higher in subjects with severe and moderate steatosis but not mild steatosis, compared with no steatosis. Although we could not detect any significant differences in the level of these different clinical parameters in subjects with mild steatosis versus no steatosis, we observed a tendency of increase in these variables' grades. We also found that the levels of clinical variables including albumin (Figure 1G), creatinine (Figure 1H), and creatine kinase (Figure 1I) as well as the amount of fat‐free mass in right arm (Figure 1J) were significantly higher in subjects with severe steatosis but not in subjects with moderate and mild steatosis, compared with no steatosis. Similarly, we observed a tendency of increase for above clinical and physical variables in subjects with moderate and mild steatosis versus no steatosis. Thus, the above eight clinical and physical variables significantly elevated in the moderate and/or severe groups were the key clinical variables during MAFLD progression. Based on these variables, we performed principal component analysis (PCA), and this showed a distinct separation between no, mild, moderate, and severe steatosis (Figure 1K).

### 2.2 Dysbiosis in the Gut and Oral Microbiome of MAFLD Patients

**Figure 2:**
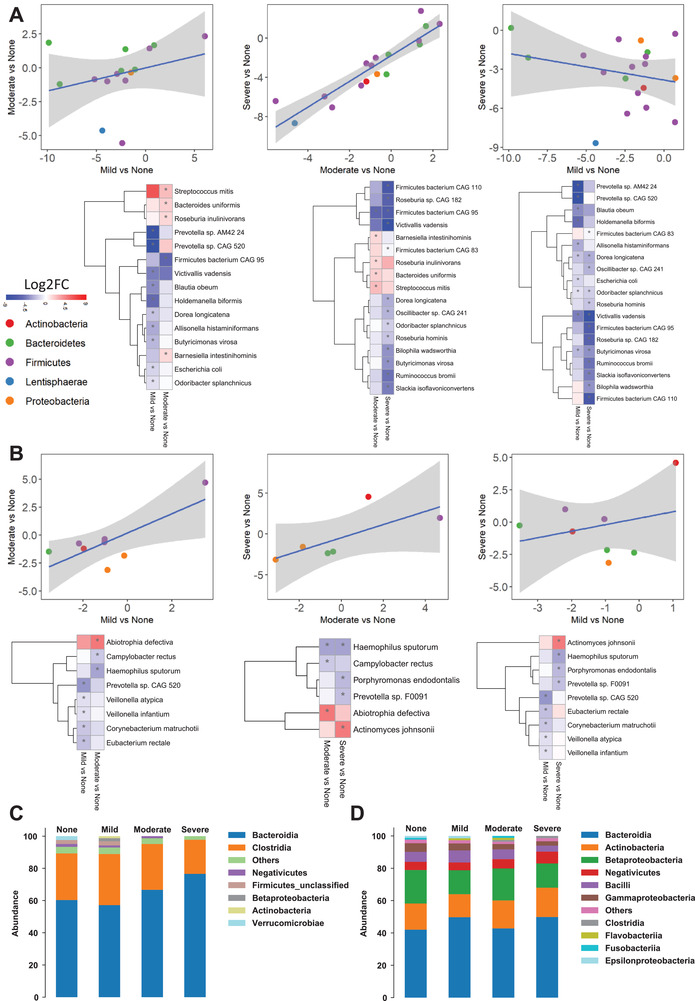
A) Scatter plot with a linear regression line, and heatmap shows Log2FC‐based alterations of the significantly different species in the A) gut microbiome and B) oral microbiome of subjects with different hepatic steatosis degrees. Asterisks indicate statistical significance based on paired Wilcoxon signed‐rank tests. *p* < 0.05. Log2FC: log2(fold change). Stacked bar plots depicting class‐level differences in C) gut microbiome and D) oral microbiome composition between the severe‐no, moderate‐no, and mild‐no steatosis groups. The “other” subcategory included viruses, fungi, and rare species (abundance <1%). The sample sizes of none, mild, moderate, and severe steatosis groups are 10, 14, 20, and 12, respectively.

We generated metagenomics data to study the dysbiosis in the microbial composition in the gut and oral microbiome (Dataset S3, Supporting Information) and explored the interactions between the host and microbiome in subjects with varying degree of HS. First, we identified the species with significant changes in the abundance of gut and oral microbiome in the mild, moderate, or severe steatosis group compared to the no steatosis group (Wilcoxon signed‐rank test, *p* < 0.05). Then, we performed a linear regression analysis for these significantly changed species between different HS groups using the Log2 fold changes (Log2FC) between each HS group versus no steatosis group. As shown in **Figure** **2**A, the change of species showed a positively correlated tendency between the mild and moderate groups and between the moderate and severe groups while a negatively correlated tendency between the mild and severe groups, suggesting a large difference of gut microbiome composition, when the status develops into the severe steatosis stage. In contrast, the species from the oral microbiome always showed a positively correlated tendency between different HS groups, suggesting a fraction of similar changes in the oral microbiome composition between different groups (Figure 2B).

Based on the differential analysis of the gut microbiome, we found that the abundances of individual species in Bacteroidetes (*Prevotella* sp CAG 520, *Prevotella* sp AM42 24, *Butyricimonas virosa*, and *Odoribacter splanchnicus*), Proteobacteria (*Escherichia coli*), Lentisphaerae (*Victivallis vadensis*), and Firmicutes (*Holdemanella biformis, Dorea longicatena, Allisonella histaminiformans*, and *Blautia obeum*) were significantly reduced in subjects with mild steatosis versus no steatosis. Notably, when we compared moderate versus no steatosis, we found that the abundance of only *Firmicutes bacterium* CAG 95 was significantly reduced. In contrast, the abundance of species belonged to Firmicutes (*Streptococcus mitis* and *Roseburia inulinivorans*) and Bacteroidetes (*Barnesiella intestinihominis* and *Bacteroides uniformis*) was significantly increased in subjects with moderate steatosis versus no steatosis (*p* < 0.05, Figure 2A and Dataset S4, Supporting Information). Moreover, we compared the species' abundances in the gut microbiome between severe versus no steatosis patients. We found that the abundances of the species in Actinobacteria (*Slackia isoflavoniconvertens*), Bacteroidetes (*Butyricimonas virosa* and *Odoribacter splanchnicus*), Lentisphaerae (*Victivallis vadensis*), Firmicutes (*Dorea longicatena, Firmicutes bacterium* CAG 83, *Firmicutes bacterium CAG 95, Firmicutes bacterium* CAG 110, *Roseburia hominis, Roseburia sp* CAG 182, *Oscillibacter sp* CAG 241, and *Ruminococcus bromii*), and Proteobacteria (*Bilophila wadsworthia*) were significantly reduced in subjects with severe steatosis versus no steatosis (*p* < 0.05, Figure 2A and Dataset S4, Supporting Information). We observed that the abundance of *Firmicutes bacterium CAG 95* was significantly reduced in the gut microbiome of subjects with both severe and moderate steatosis versus no steatosis.

Similarly, we compared the differences in the species' abundances between mild versus no steatosis in the oral microbiome. We found that the abundance of the specific species in Firmicutes (*Veillonella atypica, Veillonella infantium*, and *Eubacterium rectale)*, Bacteroidetes (*Prevotella* sp CAG 520), and Actinobacteria (*Corynebacterium matruchotii*) was significantly reduced in subjects with mild steatosis versus no steatosis (*p* < 0.05, Figure 2B and Dataset S4, Supporting Information). Our findings also revealed increased abundance of species in Firmicutes (*Abiotrophia defectiva*) and reduced abundance of Proteobacteria (*Campylobacter rectus* and *Haemophilus sputorum)* in subjects with moderate versus no steatosis (*p* < 0.05, Figure 2B and Dataset S4, Supporting Information). Notably, the abundance of species in Bacteroidetes (*Porphyromonas endodontalis* and *Prevotella* sp F0091) and Proteobacteria (*Haemophilus sputorum*) was significantly reduced, whereas the abundance of species in Actinobacteria (*Actinomyces johnsonii*) was significantly increased in the oral microbiome of subjects with severe steatosis versus no steatosis (*p* < 0.05, Figure 2B and Dataset S4, Supporting Information). We observed that the abundance of *Haemophilus sputorum* was significantly reduced in the oral microbiome of subjects with both severe and moderate steatosis versus no steatosis.

In line with the severity of steatosis, we found that Bacteroidia was the most, and Clostridia was the second most abundant bacteria in the gut microbiome composition. The Clostridia/Bacteroidia ratio is notably decreased in severe steatosis versus no steatosis (Figure 2C and Dataset S3, Supporting Information). Moreover, we found that the relative abundance of the Firmicutes and Negativicutes is reduced in severe steatosis versus no steatosis. On the other hand, the abundance of the Negativicutes was increased in severe steatosis versus no steatosis (Figure 2D and Dataset S3, Supporting Information).

### 2.3 The Associations between Metagenomics Data and Clinical Parameters

**Figure 3:**
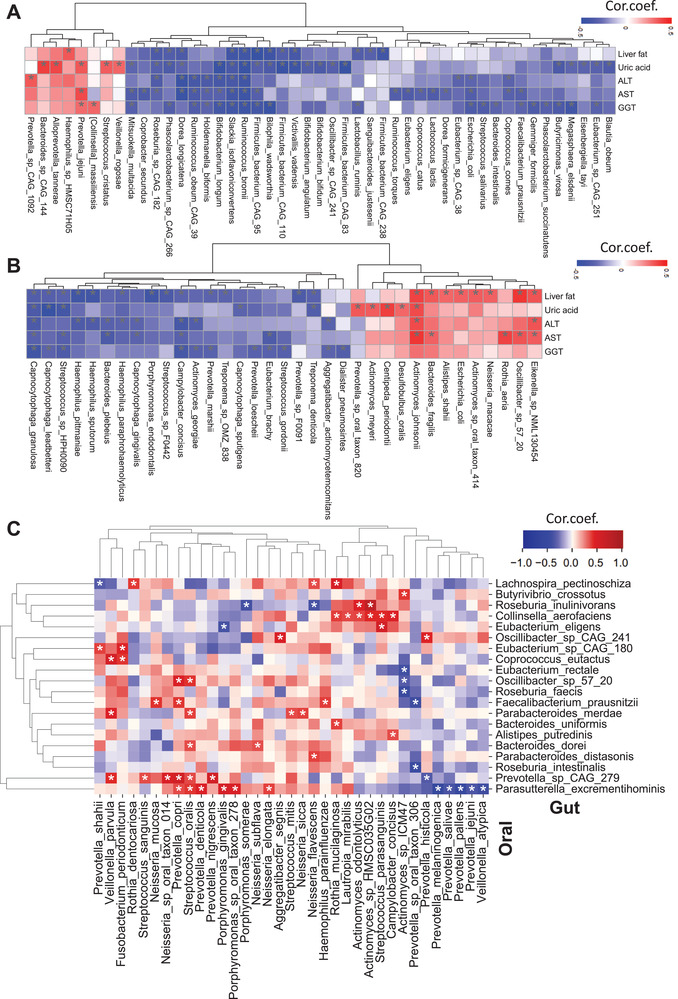
Heatmap shows the association between the five significantly different clinical variables' plasma level with the abundance of species in the A) gut microbiome and B) oral microbiome. C) Heatmap shows the association between the abundance of species in the gut and oral microbiome. Asterisks indicate the statistical significance based on Spearman correlation with *p* < 0.05. Cor.coef.: correlation coefficient. All of the 56 samples with different steatosis levels were used for the correlation analysis.

Correlation analysis between the abundances of gut and oral microbial species and the significantly altered clinical variables in both moderate and severe steatosis group showed that there are two distinct clusters with the reduced and increased abundance of species (**Figure** **3**A,B). Our analysis also showed that the abundance of species belonged to Firmicutes (*Ruminococcus bromii*, *Dorea longicatena*, and *Roseburia* sp CAG 182) was negatively correlated with HS, AST, ALT, and uric acid levels in the gut microbiome (*p* < 0.05, Figure 3A and Dataset S5, Supporting Information). Moreover, the characterization of oral microbiome revealed that the abundance of *Campylobacter concisus* and *Capnocytophaga granulosa* is negatively correlated, but *Eikenella* sp NML130454 and *Actinomyces johnsonii* is positively correlated with HS, ALT, and AST levels (Figure 3B and Dataset S5, Supporting Information). Interestingly, we found that the reduced abundance in some *Haemophilus* members was negatively correlated with the HS and ALT levels (*p* < 0.05, Figure 3B and Dataset S5, Supporting Information). Other significantly correlated species with clinical parameters are presented in the Supporting Information, Figure 3A,B, and Dataset S5 in the Supporting Information. As shown in **Table** **1**, we highlighted several gut microbial species, which were associated with the clinical variables and showed significantly different abundance at least in one of the steatosis groups compared to the none steatosis group. Meanwhile, it has been reported that these species are associated with MAFLD, dietary pattern, or immune dysregulation and contain genes which were linked with some key human metabolic pathways in Human Gut Microbiome Atlas (https://www.microbiomeatlas.org/).^[^ ^11^ ^]^ The abundance of *Ruminococcus bromii* is negatively correlated with the fibrosis severity and primary bile acid levels in nonobese MAFLD subjects.^[^ ^12^ ^]^ The abundance of *Dorea longicatena* is decreased in MAFLD‐cirrhosis^[^ ^8^ ^]^ and negatively correlated with the markers of insulin resistance in postmenopausal women with obesity.^[^ ^13^ ^]^ *Roseburia* sp CAG 182 shows higher abundance in healthy vegetarians/vegans compared to omnivores and it is related to lipid metabolism.^[^ ^14^ ^]^ *Firmicutes bacterium* CAG 95 is associated with dietary patterns and its abundance shows a strongly positive correlation with healthy plant‐ or animal‐based foods while less correlation with less healthy plant‐ or animal‐based foods.^[^ ^15^ ^]^ In addition, both *Firmicutes bacterium* CAG 95 and *Firmicutes bacterium* CAG 110 are associated with the dysregulation of fatty acid metabolism and an inflammatory surrogate GlycA in host.^[^ ^15^ ^]^ Liver fibrosis is positively correlated with the abundance of *Holdemanella biformis* in a cohort of patients with high risk for fatty liver disease.^[^ ^16^ ^]^ In addition, this species is also associated with colorectal cancer, inflammation‐related gastrointestinal diseases, and host lipid metabolism.^[^ ^17^ ^]^ The expansion of *Bilophila wadsworthia* is associated with increased inflammation, intestinal barrier dysfunction, and bile acid dysmetabolism in host, leading to higher glucose dysmetabolism and hepatic steatosis.^[^ ^18^ ^]^ The MAFLD subjects with advanced fibrosis showed increased concentration of *Escherichia coli*.^[^ ^19^ ^]^ In our analysis, we found the abundance of *Escherichia coli* and *Bilophila wadsworthia* was significantly decreased in mild or severe steatosis group compared to none steatosis group, which may be because of the different dietary patterns in different races compared to previous studies. It has been reported that the abundance of *Victivallis vadensis* is increased in high‐fat diet mouse model.^[^ ^20^ ^]^

**Table 1:** The highlighted gut and oral microbiome species, which show correlation with the clinical variables, significantly different abundance between different steatosis groups and clinical relevance

Species	Diseases or phenotypes	Reference (PMID)	Human metabolic pathways in the Human Gut Microbiome Atlas (Top three)
Gut microbiome			
*Ruminococcus bromii*	Liver fibrosis and primary bile acid metabolism in MAFLD	33020474	Glycolysis/Gluconeogenesis; Fatty acid biosynthesis; Pyrimidine metabolism
*Dorea longicatena*	Liver cirrhosis in MAFLD	28467925	Fatty acid biosynthesis; Alanine, aspartate, and glutamate metabolism; Glycine, serine, and threonine metabolism
	Insulin resistance in obesity	26075636	
*Roseburia sp CAG 182*	Higher abundance in healthy vegetarians/vegans compared to omnivores and related to lipid metabolism	34315772	–
*Firmicutes bacterium CAG 95*	Healthy dietary patterns, fatty acid metabolism, and inflammation	33432175	–
*Firmicutes bacterium CAG 110*	Fatty acid metabolism and inflammation	33432175	–
*Holdemanella biformis*	Liver fibrosis in high risk of fatty liver disease	34633706	Glycolysis/Gluconeogenesis; Pentose phosphate pathway; Galactose metabolism
	Colorectal cancer, inflammation‐related gastrointestinal diseases and host lipid metabolism	26636046	
*Bilophila wadsworthia*	Inflammation, intestinal barrier dysfunction, bile acid dysmetabolism, glucose dysmetabolism, and hepatic steatosis	30022049; 22722865; 29090023; 33482026	Glycolysis/Gluconeogenesis; Citrate cycle (TCA cycle); Pentose phosphate pathway
*Escherichia coli*	Advanced liver fibrosis in NAFLD	31484056	Glycolysis/Gluconeogenesis; Citrate cycle (TCA cycle); Pentose phosphate pathway
*Victivallis vadensis*	Increase in high‐fat diet mouse model	31847305	Glycolysis/Gluconeogenesis; Pentose and glucuronate interconversions; Galactose metabolism
Oral microbiome			
*Actinomyces johnsonii*	Periodontal infections	12102762	–
	Actinomycosis	33931097	
*Haemophilus sputorum*	Primary sclerosing cholangitis	29615776	–
*Porphyromonas endodontalis*	Periodontal infections	16390335; 12709498; 16930307	–
*Prevotella sp F0091*	Immune response	28542929	–

Similarly, we also highlighted several oral microbial species and the related diseases or phenotypes in Table 1. The abundance of *Actinomyces* contributes to the root caries, periodontal infections,^[^ ^21^ ^]^ and actinomycosis.^[^ ^22^ ^]^ The abundance of *Haemophilus* in saliva is significantly decreased in the primary sclerosing cholangitis patients compared to ulcerative colitis patients.^[^ ^23^ ^]^ *Porphyromonas endodontalis* is enriched in periodontal disease^[^ ^24^ ^]^ and *Prevotella* spp is an important pathobiont that participates in human chronic inflammation.^[^ ^25^ ^]^

### 2.4 The Link between the Oral and Gut Microbiome

We observed that the alterations in the oral microbiota correlated with the alterations in the gut microbiota. We explored the associations between the abundance of oral and gut microbiome to study host and microbiome interactions in this context. Interest in butyrate‐producing bacteria has increased with the earlier reports showing their essential role in the healthy human colon.^[^ ^26^ ^]^ Here, we found that the abundance of *Actinomyces* sp ICM47 in the oral microbiome was negatively correlated with butyrate‐producing species, namely, *Eubacterium rectale* and *Roseburia faecis* (*p* < 0.05, Figure 3C and Dataset S6, Supporting Information). Also, we found a negative correlation between the abundance of *Faecalibacterium prausnitzii* and *Roseburia intestinalis* in the gut microbiome with the abundance of *Prevotella* sp oral taxon 306 in the oral microbiome. Notably, there was a positive correlation between the abundance of *Faecalibacterium prausnitzii* in the gut microbiome with the abundance of *Haemophilus parainfluenzae, Neisseria mucosa*, and *Prevotella copri* in the oral microbiome (*p* < 0.05, Figure 3C and Dataset S6, Supporting Information). All significantly correlated species between oral and gut microbiome are presented in the Supporting Information, Figure 3C, and Dataset S6 in the Supporting Information.

### 2.5 Metabolomics Alterations in the Plasma of MAFLD Patients

To study the interactions between the microbiome and host, we generated untargeted metabolomics data based on the plasma samples of the 56 subjects and measured the abundance of 1032 metabolites (Dataset S7, Supporting Information). After excluding metabolites with missing values in >50% of samples, we analyzed the plasma level of 928 metabolites in the study (Dataset S8, Supporting Information). Then, we identified the differentially expressed metabolites between groups and revealed the key metabolites associated with the underlying molecular mechanisms related to HS progression (Dataset S8, Supporting Information).

**Figure 4:**
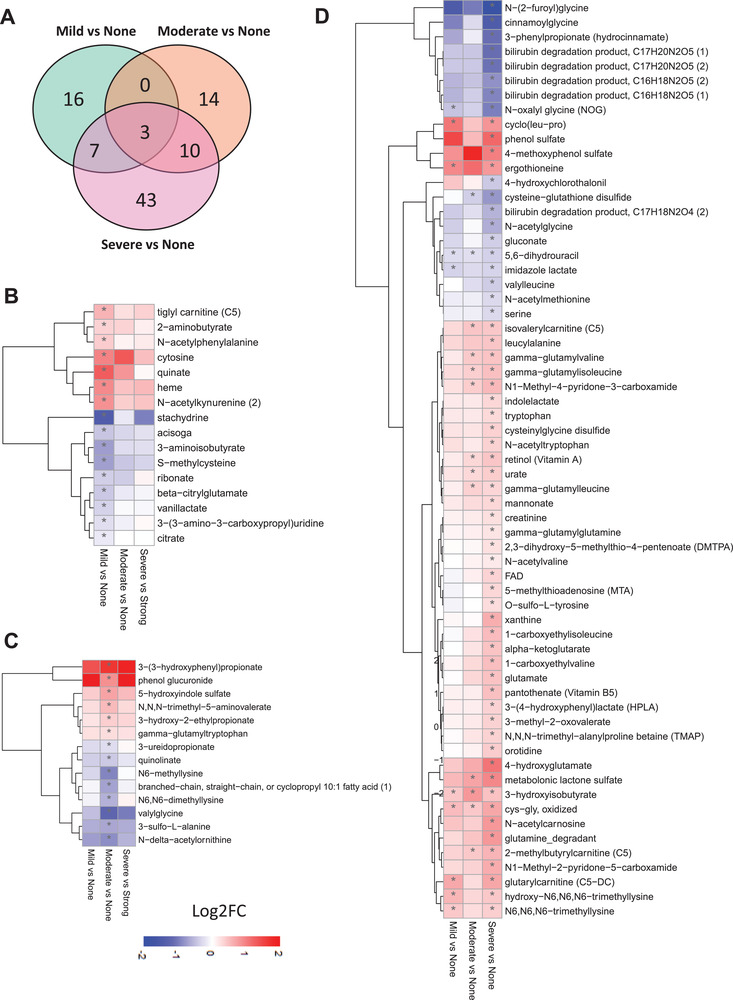
A) Venn diagram shows significantly altered nonlipid metabolites in all groups compared to none steatosis. Heatmap shows Log2FC‐based alterations of metabolites that are exclusively different in the subjects with B) mild steatosis and C) moderate steatosis compared to the subjects with no steatosis. D) All significantly altered nonlipid metabolites in the subjects with severe steatosis than the subjects with no steatosis are shown. Asterisks indicate statistical significance based on *t*‐test. *p* < 0.05. Log2FC: log2(fold change). The sample sizes of none, mild, moderate, and severe steatosis groups are 10, 14, 20, and 12, respectively.

We identified 43, 79, and 129 metabolites significantly differentially expressed in the mild, moderate, severe steatosis subject groups compared with no steatosis, respectively (Student's t‐test, *p* < 0.05, Dataset S8, Supporting Information). Of these metabolites, 17, 52, and 66 of them were associated with the lipid metabolism (Figure S1, Supporting Information) whereas 26, 27, and 63 of them were related to other parts of metabolism (e.g., amino acids, NAD+, and antioxidant metabolism) (**Figure** **4**A). Among these nonlipid metabolites, we found that 16 metabolites were significantly different only in mild steatosis versus no steatosis (Figure 4B), 14 metabolites significantly differed only in moderate steatosis versus no steatosis (Figure 4C), and 43 metabolites significantly differed only in the severe steatosis versus no steatosis (Figure 4D). We presented all 63 significantly different metabolites in the severe steatosis versus no steatosis in Figure 4D.

We found the plasma level of heme was significantly higher only in mild steatosis versus no steatosis (Figure 4B). As the precursor of pro‐ or antioxidants of biliverdin and bilirubin, the alteration of heme synthesis may be associated with increased oxidative stress in MAFLD.^[^ ^27^ ^]^ Notably, we found that bilirubin's degradation product was significantly lower in severe steatosis versus no steatosis (Figure 4D). *N*‐acetyl kynurenine, which can promote inflammation,^[^ ^28^ ^]^ also increased only in mild steatosis versus no steatosis (Figure 4B).

The plasma level of quinolinate, a precursor for nicotinamide adenine dinucleotide (NAD^+^) synthesis, was significantly downregulated only in subjects with moderate steatosis versus no steatosis (Figure 4C). It has been reported that downregulation of quinolinate is associated with MAFLD in animals,^[^ ^29^ ^]^ and the altered NAD^+^ metabolism is associated with MAFLD in humans.^[^ ^30^ ^]^ Moreover, we found that *N*,*N*,*N*‐trimethyl‐5‐aminovalerate (TMAVA) plasma level was significantly increased in moderate steatosis versus no steatosis. The increase in the plasma level TMAVA in MAFLD patients has been reported to be associated with the changes in the gut microbiome.^[^ ^31^ ^]^ It has also been proposed as a predictor of microalbuminuria in patients with type 1 diabetes.^[^ ^32^ ^]^ We found the plasma levels of serine, *N*‐acetylglycine, and glycine‐conjugated metabolites were significantly decreased only in subjects with severe steatosis versus no steatosis (Figure 4D). Previously, we have found that MAFLD is associated with serine deficiency and reported that serine and glycine are key metabolites for glutathione synthesis,^[^ ^33^ ^]^ which is required for preventing the accumulation of intermediate products of fatty acid oxidation.^[^ ^30^ ^]^ We have proposed that serine supplementation may treat these patients.^[^ ^34^ ^]^ Meanwhile, we found all the bilirubin degradation products were downregulated only in severe steatosis versus no steatosis. Bilirubin can function as an antioxidant, reducing the HS accumulation.^[^ ^35^ ^]^ On the other hand, we found that plasma level of metabolites involved in tryptophan, lysine, and uric acid metabolism was significantly increased in subject with severe steatosis (Figure 4D). We observed that the plasma level of uric acid and xanthine involved purine metabolism was significantly increased in subjects with severe steatosis versus no steatosis. We also found that the plasma level of *N*,*N*,*N*‐trimethyl‐alanylproline betaine (TMAP) associated with urea cycle was significantly increased in subjects with severe steatosis versus no steatosis. These results agree with the previous studies, where plasma uric acid level is significantly associated with HS in MAFLD patients.^[^ ^36^ ^]^

We also found that the plasma level of cysteine‐glutathione disulfide, a glutathione and cysteine‐conjugate product, was significantly lower in subjects with severe and moderate steatosis versus no steatosis. It has been reported that the concentration of cysteine‐glutathione disulfide was significantly lower in subjects with steatosis and NASH.^[^ ^37^ ^]^ We observed that plasma level of 3‐hydroxyisobutyrate (3‐HIB), oxidized cys‐gly was significantly higher and of 5,6‐dihydrouracil was significantly lower in all the three steatosis groups versus no steatosis (Figure 4D). Previously, we measured plasma levels of 3‐HIB, involved in branched‐chain amino acids (BCAAs) metabolism in around 10 000 extensively phenotyped individuals, and identified 3‐HIB as a marker of insulin resistance, mitochondrial dysfunction, and future risk of developing T2D,^[^ ^38^ ^]^ which are closely linked to MAFLD. The oxidized form of l‐cysteinylglycine is involved in glutathione metabolism, which has been reported to have a critical role in MAFLD's progression and treatment.^[^ ^4^, ^10^, ^30^, ^38^ ^]^ The reduction of uracil produces 5,6‐dihydrouracil, and it is involved in pyrimidine and beta‐alanine metabolism.

### 2.6 Associations between the Plasma Metabolome and Patient Phenotype

**Figure 5:**
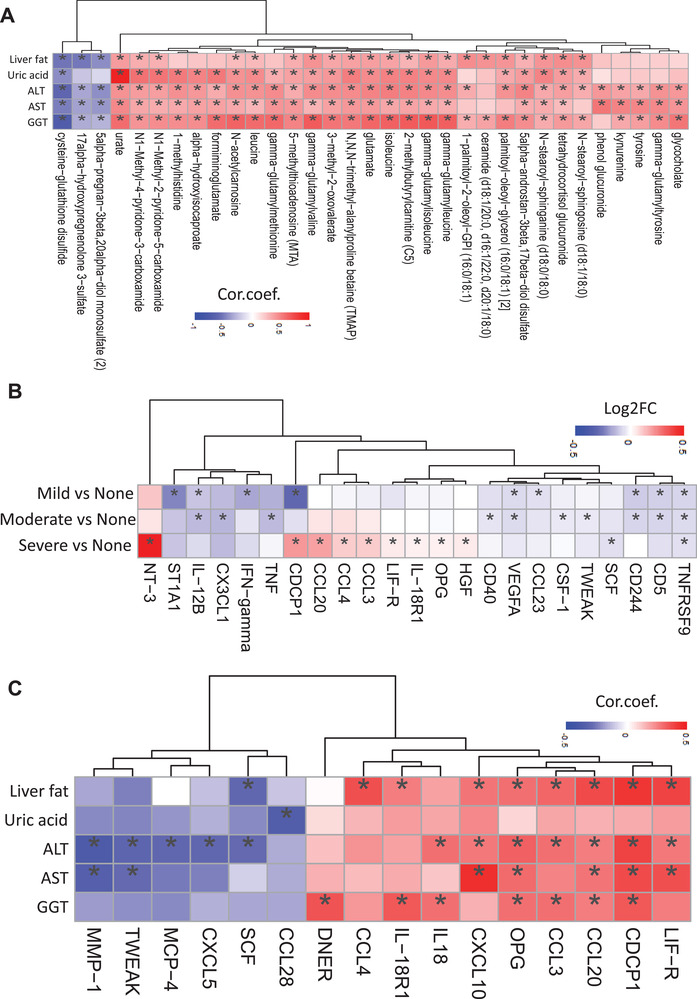
A) Heatmap shows the association between the five significantly different clinical variables' plasma level with the top 10 most significantly correlated metabolites' plasma level. Asterisks indicate the statistical significance based on Spearman correlation with *p* < 0.05. B) Heatmap shows the Log2FC‐based alterations of all the significantly altered inflammation‐related proteins in the subjects with mild, moderate, and severe steatosis compared to the subjects with no steatosis. Asterisks indicate the statistical significance based on *t*‐test with *p* < 0.05. C) Heatmap shows the association between the plasma level of the five significantly different clinical variables with the inflammation‐related proteins. Asterisks indicate the statistical significance based on Spearman correlation. *p* < 0.05. Log2FC: log2(fold change). Cor.coef.: correlation coefficient. All of the 56 samples with different steatosis groups were used in the correlation analysis.

We assessed the associations between the plasma level of significantly different five clinical parameters including liver fat, uric acid, ALT, AST, and GGT with the plasma level of metabolites (**Figure** **5**A and Dataset S9, Supporting Information). We found that all these clinical variables were positively correlated with the plasma level of TMAP and BCAA and its conjugates, including leucine, isoleucine, gamma‐glutamyl leucine, gamma‐glutamyl isoleucine, gamma‐glutamyl valine, 3‐methyl‐2‐oxovalerate, and *N*‐acetyl carnosine. Like 3‐HIB, other BCAAs products have been associated with MAFLD progression, insulin resistance, mitochondrial dysfunction, and incidence of T2D.^[^ ^39^ ^]^ The level of ALT, AST, and GGT was positively correlated with the plasma level of kynurenine, can activate inflammatory response, and has been associated with MAFLD.^[^ ^28^ ^]^ Moreover, uric acid's plasma level was significantly positively correlated with all these parameters.

In contrast, cysteine‐glutathione disulfide's plasma level was significantly negatively correlated with these five clinical parameters. The plasma level of 17alpha‐hydroxypregnenolone 3‐sulfate and 5alpha‐pregnan‐3beta,20alpha‐diol monosulfate (2) was significantly negatively associated with HS, ALT, AST, and GGT levels (Figure 5A). The differences in these metabolites' plasma level can be used to detect HS and explore the effect of treatment in MAFLD patients.

### 2.7 The Influence of the Microbiome on the Plasma Metabolome

In the gut microbiome, the abundances of the numerous species belonged to Bacteroidales (except *Provetolla copri*) and Clostridiales (except *Eubacterium rectale*) are negatively correlated with specific peptides and amino acids (e.g., gamma‐glutamyl‐alpha‐lysine, S‐adenosylhomocysteine, gamma‐glutamyl threonine, and threonine) (Figure S2, Dataset S13, Supporting Information). The metabolites associated with primer and seconder bile acid metabolism are negatively correlated with abundances of the *Roseburia intestinalis, Parabacteriodes diastonis, Bacteroides vulgatus*, and *Bacteroides uniformis* (Figure S2, Dataset S13, Supporting Information). Of note, these species showed a positive correlation with isoursodeoxycholate, associated with secondary bile acid metabolism (Figure S2, Dataset S13, Supporting Information).

In the oral microbiome, the correlation between the abundances of the individual species and significantly altered plasma metabolites in different HS groups was positively correlated with the abundances of certain species in Bacteroidia and *Neisseria subflava* but negatively correlated with the abundances of the *Actinomyces* spp. and *Rothia dentocariosa* (Figure S3, Dataset S13, Supporting Information). Moreover, we observed that the abundance of *Campylobacter concisus* and *Veillonella atypica*, both were significantly negatively correlated with steatosis, was also associated with the plasma level of metabolites involved in carnitine metabolism (Figure S3, Dataset S13, Supporting Information). All associations between the plasma level metabolites and the abundance of the species in the gut and oral microbiome are presented in the Supporting Information, Figure S3, and Dataset S13 in the Supporting Information.

### 2.8 Inflammatory Proteomics Alterations in the Plasma of MAFLD Patients

Plasma levels of 94 inflammatory protein markers were measured by the proteome profiling platform proximity extension assay (PEA). After quality control and exclusion of proteins with missing values in more than 50% of samples, 72 proteins were analyzed (Dataset S10, Supporting Information). Proteins whose expression levels significantly differed between groups are presented in Dataset S11 in the Supporting Information.

Except the plasma level of CDCP1 which is significantly lower in mild steatosis and significantly higher in severe steatosis versus no steatosis, majority of the proteins followed the same directional changes in all steatosis groups (Figure 5B). It has been reported that CDCP1 knockout mice have increased lipid accumulation in the liver^[^ ^40^ ^]^ which may explain the downregulation of CDCP1 in mild steatosis compared with no steatosis in our study. Besides, CDCP1 also acts as a profibrotic mediator which may play a central role in subjects with severe steatosis and fibrosis.^[^ ^41^ ^]^

We found that the plasma level of TNFRSF9 was significantly lower in all three steatosis groups versus no steatosis (Figure 5B). It has been reported that stimulation of TNFRSF9 with agonistic antibody reduces adiposity, body weight, and HS and increases energy expenditure in diet‐induced obese mice and genetically obese/diabetic mice.^[^ ^42^ ^]^ The plasma level of ST1A1, IFN‐gamma, and CCL23 was lower only in mild steatosis versus no steatosis (Figure 5B). The differences in the mRNA expression of ST1A1 are associated with high‐fat diet‐induced obesity. The plasma levels of CX3CL1, TNF, CD40, CSF‐1, and TWEAK were lower in moderate steatosis versus no steatosis (Figure 5B). The downregulation of CX3CL1/CX3CR1 pathway has been suggested as a mechanism underlying *β* cell dysfunction in type 2 diabetes.^[^ ^43^ ^]^ It has been reported that TNF level contributes HS in diet‐induced obesity,^[^ ^44^ ^]^ CD40 deficiency in mice exacerbates obesity‐induced HS and insulin resistance,^[^ ^45^ ^]^ mice lacking CSF‐1 have reduced adiposity,^[^ ^46^ ^]^ and decreased serum level of TWEAK concentration is associated with the MAFLD.^[^ ^47^ ^]^

The plasma level of NT‐3, CCL20, CCL4, CCL3, LIF‐R, OPG, and HGF was higher, and SCF was lower only in the severe steatosis versus no steatosis (Figure 5B). It has been shown that the protein level of CCL20 was increased in MAFLD.^[^ ^48^ ^]^ An increased mRNA expression of LIF‐R has been demonstrated in high‐fat diet‐induced MAFLD mice.^[^ ^49^ ^]^ Moreover, increased circulating levels of HGF have been reported in NASH.^[^ ^50^ ^]^

Then we assessed the associations of the significantly differed five clinical variables with the plasma levels of the inflammation‐related proteins (Figure 5C and Dataset S12, Supporting Information). We identified two main clusters. These variables were negatively and positively correlated with at least one of the inflammation‐related proteins in the first and second clusters, respectively.

### 2.9 The Influence of the Microbiome on the Plasma Proteome

In the gut microbiome, we found that the abundances of *Coprococcus eutactus*, *Dialister* sp CAG 357, *Oscillibacter* sp 57 20, and *Eubacterium* sp CAG 180 were most positively associated with the inflammatory protein levels (Figure S4, Dataset S14, Supporting Information). On the other hand, the abundances of *Roseburia intestinalis*, *Eubacterium eligens*, *Parabacteroides distasonis*, *Roseburia faecis*, *Butyrivibrio crossotus*, and *Prevotella copri* were negatively correlated with the plasma levels of inflammation‐related proteins (Figure S4, Dataset S14, Supporting Information). Of note, the IL10 plasma level was positively correlated with the abundances of *Collinsella aerofaciens* and *Alistipes finegoldii* but negatively correlated with the abundances of *Roseburia intestinalis*, a primary degrader of dietary fiber^[^ ^26^ ^]^ (Figure S4, Dataset S14, Supporting Information). In the group with the moderate steatosis, we found that the abundances of *Coprococcus eutactus* are positively correlated with the levels of the inflammatory proteins that are significantly altered in subjects with severe steatosis.

In the oral microbiome, we found some species within *Neisseria*, *Rothia*, and *Veillonella* were positively associated with the numerous inflammatory proteins (Figure S5, Dataset S14, Supporting Information). However, there was a negative correlation between the abundance of species belonging to the *Porphyromonas* and the *Prevotell*a with the inflammation‐related proteins (Figure S5, Dataset S14, Supporting Information). Interestingly, the abundances of the *Neisseria flavescens, Haemophilus parainfluenzae*, and *Campylobacter concisus* were also negatively correlated with inflammation‐related proteins (Figure S5, Dataset S14, Supporting Information). Besides, FGF‐21 plasma level was negatively correlated with the abundances of *Streptococcus mitis* and *Tannerella* sp oral taxon HOT 286 and IL‐6 plasma level was negatively correlated with the abundances of *Porphyromonas endodontalis* (Figure S5, Dataset S14, Supporting Information). Other significantly correlated species with plasma inflammation‐related proteins are presented in the Supporting Information, Figure S5, and Dataset S14 in the Supporting Information.

### 2.10 Prediction of HS based on Multiomics Data

**Figure 6:**
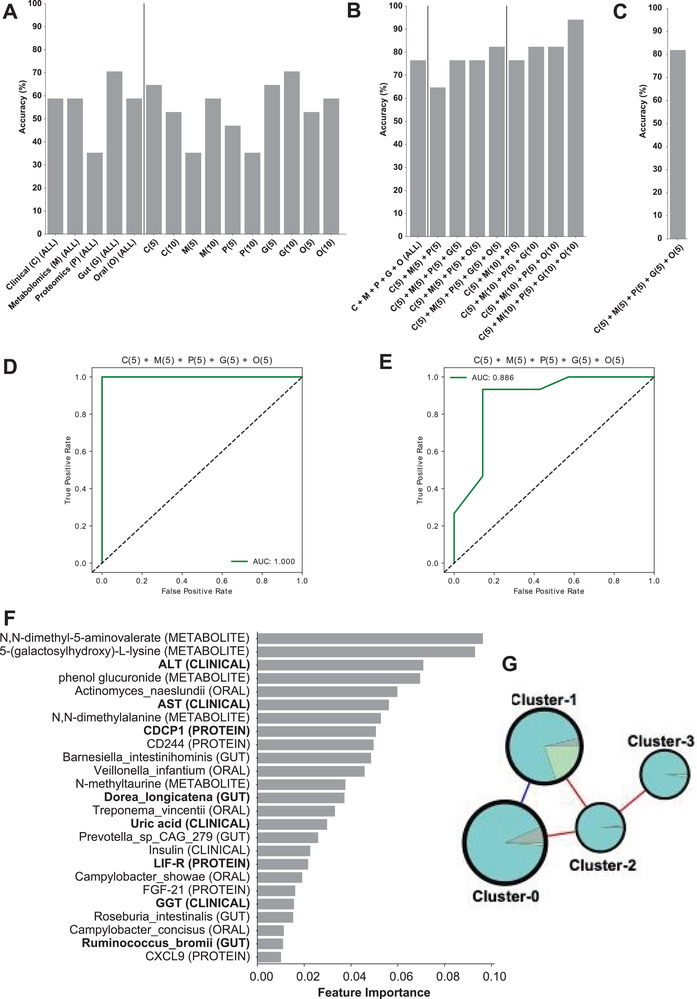
Accuracy score of random forest classification algorithm performed to predict the class using A) single omics and B) multiomics combination of top features from each omics type in finding (56 subjects) and C) validation (22 subjects) of datasets. Numbers in the brackets represent the number of top features taken from the single omics data. D) AUC‐ROC curve for prediction of HS based on the data from 56 subjects. E) AUC‐ROC curve for validation of the final model in prediction of HS based on the data from 22 subjects. F) Top 25 features from the model with the highest accuracy, C(5) + M(5) + P(5) + G(5) + O(5). Analytes that are altered significantly between the groups are marked in bold. G) Composition of each cluster in the multiomics network.

We analyzed phenomics, metabolomics, proteomics, and oral/gut metagenomics data and identified features differentiating between the groups of subjects with varying HS degrees in the overall cohort with 56 subjects. We performed analyses using single/multiomics data using the Random Forest algorithm and discovered the key features associated with HS. First, we used all individual data points from each omics data (**Figure** **6**A and Figures S6 and S7, Dataset S15, Supporting Information). We found that the gut metagenomics data were the top‐performing dataset in the prediction of the steatosis degree, with > 70% accuracy (area under the curve (AUC): 0.90) (Figure 6A). In contrast, we found that the inflammatory proteomics data were the worst‐performing data, with only 35.3% accuracy (AUC: 0.72) (Figure 6A). Next, we tested the top five or ten features from each omics dataset and tried their different combinations (Figure 6B and Dataset S15 and Figure S8A–H, Supporting Information). We observed that the varieties of top features in clinical (five variables), metabolomics (ten variables), and proteomics (five variables) yielded 64.7% accuracy (AUC: 0.92) (Figure S8A–F, Supporting Information), whereas adding gut and oral metagenomics (ten top features each) showed the highest accuracy of 94.1% (AUC: 0.951) in the prediction of the steatosis degree (Figure 6B and Figure S8G–I, Supporting Information). In the follow‐up cohort with 22 subjects, the model yielded 82.7% accuracy (AUC: 0.843) by adding these top features in clinical (five variables), metabolomics (ten variables), and proteomics (five variables), and gut and oral metagenomics (ten top features each) (Figure 6C and Figure S8J, Supporting Information). We had the highest predictivity of HS (AUC: 1.0) by adding top five features in clinical, metabolomics, proteomics, gut metagenomics, and oral metagenomics data in the finding dataset (Figure 6D). We also validated the predictivity of the final model with 22 subjects and found that the model yielded a higher predictivity AUC: 0.886) (Figure 6E) with the same key features compared to other combinations (Figure S8J, Supporting Information).

The top five features in clinical (ALT, AST, uric acid, insulin, and GGT), metabolomics (*N*,*N*‐dimethyl‐5‐aminovalerate, 5‐(galactosylhydroxy)‐L‐lysine, phenol glucuronide, *N*,*N*‐dimethylalanine, and *N*‐methyltaurine), proteomics (CDCP1, CD244, LIF‐R, FGF‐21, and CXCL9), gut metagenomics (*Barnesiella intestinihominis, Dorea longicatena, Prevotella* sp CAG 279, *Roseburia intestinalis*, and *Ruminococcus bromii*), and oral metagenomics (*Actinomyces naeslundii, Veillonella infantium, Treponema vincentii, Campylobacter showae*, and *Campylobacter concisus*) used in prediction of HS can be considered as candidate biomarkers for MAFLD (Figure 6F and Dataset S15, Supporting Information). In our study, we also found that the abundance of the gut microbiome species including *Barnesiella intestinihominis, Dorea longicatena*, and *Ruminococcus bromii* and oral microbiome species including *Campylobacter concisus* and *Veillonella infantium* was significantly associated with HS and reported that their abundance was significantly correlated with the plasma level of metabolites and inflammatory proteomics levels.

Some of these markers have been reported and validated in other studies. In our previous study,^[^ ^51^ ^]^ we found that the level of ALT, AST, Insulin, CDCP1, and FGF‐21 were significantly positively correlated with the various ectopic fat depots, including HS. Moreover, the abundance of gut microbiome species, including *Dorea logicatena* and R*uminococcus bromii*, has been reported as possible markers to predict HS.^[^ ^12^, ^13^ ^]^ The abundance of oral microbiome species, including *Porphyromonas endodontalis* and *Campylobacter concisus*, was also significantly negatively correlated with the HS based on previous studies.

### 2.11 Integrative Analysis of Multiomics Data using Biological Networks

We generated an integrative multiomics network for showing the relationships between different analytes within and between other omics datasets. The network was built using Spearman correlation analysis (Dataset S16, Supporting Information), between all analytes from the aforementioned omics data, filtered by edges with FDR < 0.05, resulting in a relatively sparse network with 1032 nodes and 17 536 edges (3.3% network density). The complete network is presented in iNetModels (http://inetmodels.com), an interactive multiomics networks database and visualization. We performed a centrality analysis on the network by calculating each node's degree (Dataset S16, Supporting Information). We found that the top 20 most connected nodes were related to lipid metabolism, e.g., ceramides, sphingomyelins, diacylglycerol, and phospholipid‐related sub‐pathways. Similarly, we found lipid‐related clinical variables as top nodes in clinical variables (total cholesterol, GGT, LDL, and TG‐level). In contrast, top inflammatory proteomics nodes were STAMBP, TNFSF14, SIRT2, CXCL5, CXCL1, and CD40, associated with cytokine–cytokine receptor interaction, and NF‐kappa B, TNF, and IL‐17 which are associated with several signaling pathways.

Subsequently, we performed a clustering analysis using the Leiden community analysis algorithm. We then combined the smaller clusters (11 clusters with 1–54 analytes) to the next biggest cluster (cluster‐3) to balance the cluster size, resulting in four clusters to use in further analysis (Figure 6G and Dataset S16, Supporting Information). We found that all clusters had positive correlations with each other, except the connection between cluster‐0 and cluster‐1 (Figure 6G), based on their shared edges. Looking at the central analytes in each cluster (Dataset S14, Supporting Information), as expected due to the unbalanced number of analytes in the clusters, metabolites dominate each cluster's top analytes. In cluster‐0, metabolites related to amino acid metabolisms were on top, specifically isoleucine and its derivative and gamma‐glutamyl amino acids, together with a clinical variable, GGT, whereas cluster‐1 was dominated by phospholipid, carbohydrate, and taurine metabolism, followed by the central proteins. Top 20 most connected metabolites and top clinical variables mentioned above, were concentrated in cluster‐2, which contained most lipid‐related metabolites and clinical variables, making this cluster as candidate central variables in HS (Dataset S16, Supporting Information). Finally, we found that cluster‐3 contained mostly metabolites related to fatty acid metabolism. Interestingly, the clustering analysis showed sub‐networks with analytes, which has similar functionality; this shows the power of biological networks in establishing the functional relationships between analytes based on the multiomics analysis.

**Figure 7:**
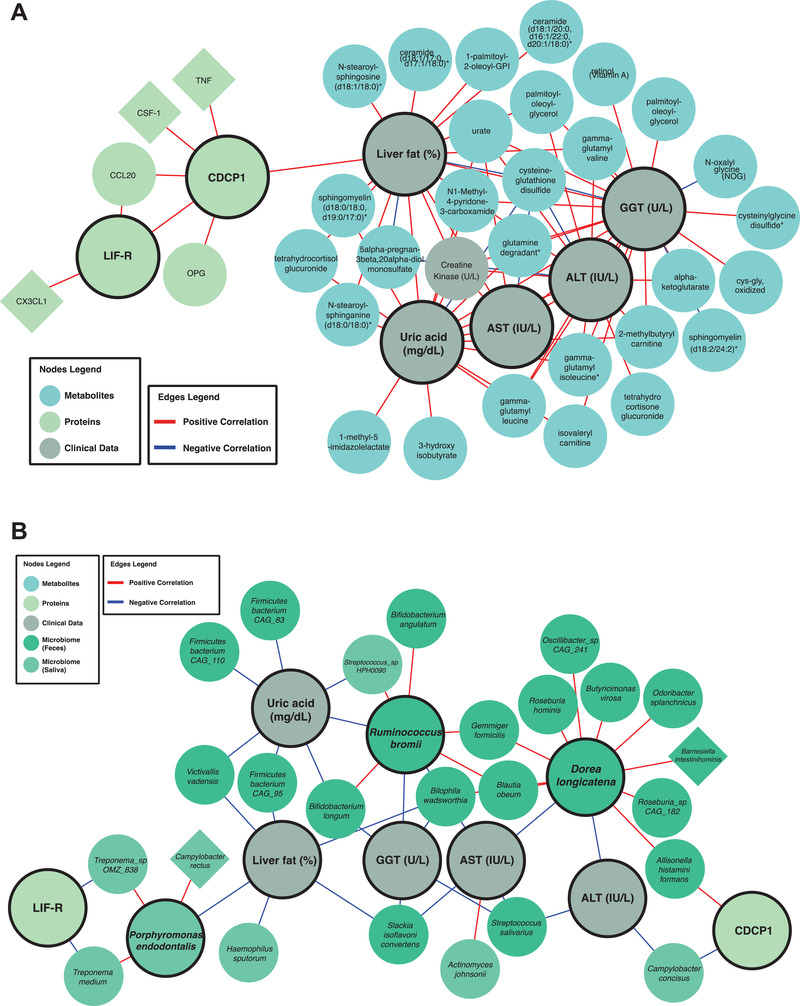
Significantly different first neighbors of significantly different analytes in the best‐performing random forest model in the A) multiomics and B) metagenomics‐centric correlation networks.

We took the features from our multiomics random forest model and filtered only the significantly altered analytes, resulting in nine analytes fulfilling those requirements (ALT, AST, GGT, uric acid, CDCP1, LIF‐R, *Dorea longicatena* (gut), *Ruminococcus bromii* (gut), *Porphyromonas endodontalis* (oral)). Next, we retrieved the subnetwork with the first neighbor of those features as well as HS and overlaid comparative analysis results (filtering *p* < 0.05) (**Figure** **7**A). We found that those key features related with HS, and their first neighbors were dominated by lipid metabolites, gamma‐glutamyl amino‐acids, BCAA metabolites, fatty acid metabolism (carnitine derivatives), and glutathione‐related metabolites that are significantly associated with the HS.

Metagenomics data were not included in the multiomics network built previously due to the data's sparsity that caused dissociation of the network hence reducing the network analysis's power. We decided to construct a metagenomics centric network, by including the key features from random forest analyses and all microbial species with > 1% abundance in at least five samples (Figure 7B). We have integrated the complete networks into iNetModels platform. Similar to the previous approach, we overlapped the comparative analysis results (*p* < 0.05) and retrieved the sub‐network. HS was significantly negatively correlated with the abundance of *Victivallis vadensis, Firmicutes bacterium* CAG 95, *Slackia isoflavoniconvertens*, and *Bilophila wadsworthia* in the gut, and *Porphyromonas endodontalis* and *Haemophilus sputorum* in the oral microbiome. Interestingly, the abundance of *Slackia isoflavoniconvertens* and *Bilophila wadsworthia* in the gut also negatively correlated with HS, AST, and GGT levels. Another key species in the gut microbiome is *Streptococcus salivarius*, whose abundance was negatively correlated with ALT, AST, and GGT levels. On the other hand, the abundance of *Dorea longicatena* was negatively correlated with both ALT and AST levels and positively correlated with numerous members of Bacteroidales and Clostridiales. We also found that the abundance of *Campylobacter concisus* is associated with levels of both ALT and CDCP1, a transmembrane receptor associated with aggressive epithelial cancers. Additionally, we observed a positive correlation between AST levels and the abundance of *Actinomyces johnsonii* in the oral microbiome. We also observed that abundance of *Ruminococcus bromii* was significantly positively associated with the level of uric acid and GGT. Uric acid levels are also negatively correlated with the abundance of specific species in Firmicutes phylum and *Bifidobacterium longum* and *Victivallis vadensis*. Based on these results, the integrative network analysis with multiomics data strengthened the results from single omics analyses and added additional power to identify key features associated with HS. Moreover, it allowed us to reveal functional relationships within and between different omics data.

## 3 Discussion

**Figure 8:**
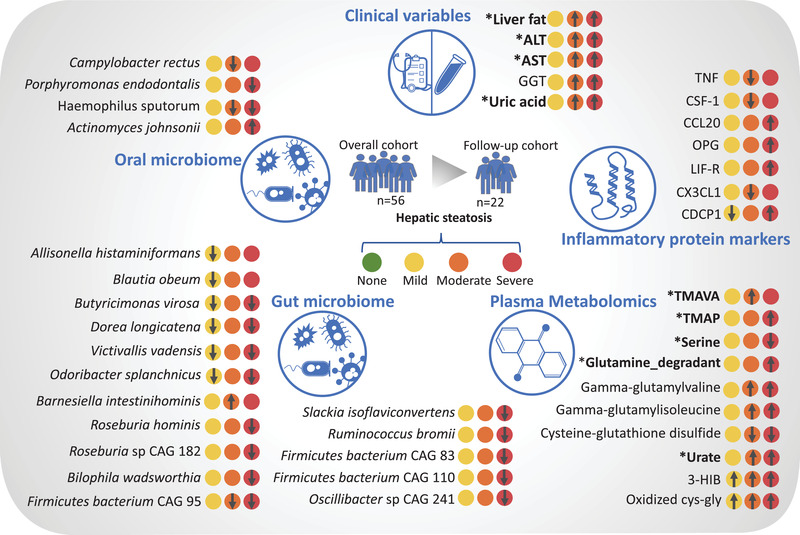
The key changes in phenomics, oral and gut microbiome, plasma metabolomics, and plasma proteomics. These analytes are shown since they are significantly changed at least in one of the HS group compared to the no steatosis group. These parameters correlated with other omics data based on the network analysis. The arrow represents the significant upregulation or downregulation of a variable in a specific HS group compared with no steatosis group. * represents that the level of the analyte could be reversed after the supplementation of CMAs in our recent MAFLD clinical phase 2 study.

In this study, we presented the key findings of our multiomics analysis in MAFLD patients (**Figure** **8**). In phenomics, we found five main phenotypes liver fat, ALT, AST, uric acid, and GGT which were significantly elevated in moderate and severe steatosis groups compared to the no steatosis group (Figure 8). In our recent MAFLD clinical phase 2 study, we successfully reduced the liver fat, ALT, AST, and uric acid by the supplementation of combined metabolic activators (CMAs).^[^ ^52^ ^]^

The microbial imbalance has been identified as a key player in MAFLD's pathogenesis due to the functional crosstalk between liver and complex microbial composition.^[^ ^10a^ ^]^ Microbiota can improve or aggravate liver diseases through several mechanisms, including enhanced liver lipid metabolism, elevated alcohol production, altered energy metabolism, impaired intestinal permeability, and disrupted bile secretion.^[^ ^53^ ^]^ Previous studies demonstrated that MAFLD patients had higher TNF‐alpha and IL‐6 in the mucosal layer of the intestinal wall^[^ ^54^ ^]^ and reported larger quantities of pathogenic bacteria in the gut.^[^ ^55^ ^]^

Our study used shotgun sequencing (enabling enhanced taxonomic resolution) of saliva and feces samples to analyze the composition of oral and gut microbiota. We showed that these diverse communities are associated with different steatosis levels in a well‐characterized overweight and obese MAFLD cohort. In the gut microbiome, we identified significant alterations in certain species following the existing literature; emphasizing that the abundances of *Dorea longicatena* were reduced in patients with steatosis, and the abundances of *Slackia isoflavoniconvertens, Roseburia hominis*, and *Ruminococcus bromii* were reduced in severe steatosis. Some of these species have been proposed to be essential for healthy microbiota homeostasis previously. The abundances of *Dorea longicatena* have been found to be reduced in MAFLD‐cirrhosis,^[^ ^8^ ^]^ negatively correlated with the markers of insulin resistance in postmenopausal women with obesity,^[^ ^13^ ^]^ and exhibited higher levels in remission of Crohn's disease.^[^ ^56^ ^]^ The abundances of *Slackia isoflavoniconvertens*, are an equol producer bacteria by conversion of the soy isoflavone, have been endorsed as having many favorable effects on the host metabolism;^[^ ^57^ ^]^ *Ruminococcus bromii* is another beneficial species for human health, and its abundance was inversely correlated with the fibrosis severity and primary bile acid levels in nonobese MAFLD subjects. An interventional animal study suggested a potential role in synthesizing secondary bile acids.^[^ ^12^ ^]^ Moreover, the abundance of *Barnesiella intestinihominis* was found to be significantly overrepresented in the stool with a potency to induce MAFLD based on 16S rDNA profiling of mice.^[^ ^58^ ^]^

Generation of the metabolomics and proteomics data allowed for studying the molecular pathways and identifying key features associated with the MAFLD progression. We observed that the metabolites involved in the glutathione metabolism, BCAA metabolism, and pyrimidine metabolism, which are the key pathways in MAFLD, were already altered from HS's early stage. In the patients with moderate steatosis, we identified elevated TMAVA in moderate steatosis group, a biomarker used to predict gut microbiome change, confirming that TMAVA may be an essential feature of MAFLD.^[^ ^59^ ^]^ In our MAFLD clinical phase 2 study, TMAVA was significantly reduced by the supplementation of CMAs.^[^ ^52^ ^]^ We found that plasma level of TMAVA was significantly positively correlated with the abundance of *Bacteroides stercoris*, *Bacteroides uniformis*, *Parabacteroides distasonis* and negatively correlated with the abundance of *Prevotella copri* in the gut microbiome. The TMAVA plasma level is also significantly negatively correlated with the abundance of the *Veillonella dispar* and *Veillonella atypica*, one of the key species associated with HS, in the oral microbiome. Notably, we identified *N*,*N*‐dimethyl‐5‐aminovalerate (di‐methylated forms TMAVA) as one the most critical feature in the prediction of MAFLD.

Moreover, we observed that serine and glycine‐related metabolites were altered in the severe stage of steatosis, further highlighting their crucial roles in MAFLD.^[^ ^30^, ^33^ ^]^ Serine, one of the critical component in our CMAs and the glycine‐related metabolites (e.g., N‐acetylglycine) were significantly increased after the supplementation of CMAs.^[^ ^52^ ^]^ Besides, we observed that heme, the precursor of antioxidant of bilirubin, and bilirubin degradation products were altered in mild and severe steatosis compared with no steatosis, respectively. This suggests that the redox balance may be changed at the early stage of HS. Based on the comparison of proteomics data of different degrees of steatosis versus no steatosis, we observed a decreasing tendency of most inflammation‐related proteins in mild and moderate versus no steatosis but an increasing trend in the severe steatosis versus no steatosis.

More commonly, the liver immune tolerance mechanism, processing immunosuppressive functions by regulating cytokines or chemokines' expression, limits the magnitude of intrahepatic immune responses and allows the liver to recover.^[^ ^60^ ^]^ However, immune tolerance is broken by the further accumulation of fat, which induces severe steatosis. As a result, enhanced antigen presentation to lymphocytes associated with the increased expression of inflammation‐related proteins leads to the development of both cellular and humoral immune responses.^[^ ^61^ ^]^ Besides, we observed that the abundance of the species, including *Barnesiella intestinihominis*, *Oscillibacter* sp CAG 241, and *Roseburia inulinivorans* associated with HS was significantly correlated with the inflammatory proteomics plasma levels. We also observed that the abundance of HS‐associated species including *Campylobacter concisus* (negatively correlated with CXCL9), *Porphyromonas endodontalis* (negatively correlated with LIF‐R), and *Veillonella atypica* (positively correlated with CD244) in the oral microbiome was significantly correlated with the plasma level of the inflammatory proteomics plasma levels.

A major limitation of this study is that the subjects we recruited had similar BMI levels. Thus, it is expected that the waist levels are not significantly different between the steatosis groups. In addition, we did not observe any significant difference for the glucose and insulin levels between the different steatosis groups. It may be because the subjects from none and/or mild steatosis groups have higher variation of levels of glucose and insulin than the subjects from moderate and severe steatosis groups (Table 1). Interestingly, the levels of albumin and right arm fat free mass are significantly increased in mild and/or severe groups, which is worthwhile to further study its potential mechanism. We would like to expand the overall sample size in our future work, which may provide the possibility to analyze the association of hepatic steatosis with different omics within different BMI, glucose tolerance, and age levels as well as different genders.

In conclusion, we performed a multiomics analysis of subjects with varying degrees of HS and integrated these data using systems approaches to identify HS's key features. We revealed the alterations in the microbial compositions start at early stages of the clinical spectrum and cause metabolic disturbances underlying HS. We also studied the effect of these alterations on the host metabolism by performing plasma metabolomics, and inflammatory proteomics analysis. Hence, we revealed the underlying molecular mechanisms involved in the progression of HS. We envisage that our results can be used to discover prognostic and predictive clinical markers and develop efficient therapeutic strategies.

## 4 Experimental Section

### Participants

Overweight or obese patients 18–70 years of age were enrolled in the trial if they were diagnosed with MAFLD and met all the inclusion criteria: BMI >27 kg m^−2^, triglycerides ≤354 mg dL^−1^, low‐density lipoprotein cholesterol ≤175 mg dL^−1^, and increased HS (>5.5%). Patients were excluded if they carried the PNPLA3 I148M (homozygous for I148M), had ALT or AST levels >threefold higher than the upper limit of normal, or had taken oral antidiabetics, including metformin, within 3 months. The main characteristics of the study participants are presented in Dataset S1 in the Supporting Information.

MRI‐PDFF determined HS, and plasma samples for proteomics and metabolomics analyses were collected (Dataset S1, Supporting Information). Patients for this characterization study were recruited at the Koç University Hospital, Istanbul, Turkey (Dataset S1, Supporting Information). The study was conducted following Good Clinical Practice guidelines and the principles of the Declaration of Helsinki. An independent external data monitoring committee oversaw the safety of the participants and the risk‐benefit analysis. Written informed consent was obtained from all participants before trial‐related procedures were initiated. The Koç University ethics committee approved the study (Decision no: 2018.351.IRB1.043, Decision Date: 15 May 2019).

### Metagenomics Data Analysis

Fresh stool and saliva specimens were collected and preserved using DNA/RNA Shield Fecal Collection tubes (Zymo Research, Irvine, CA) and DNA/RNA Shield Collection Tube (Zymo Research, Irvine, CA), respectively. DNA extractions from the fecal samples were done using QIAamp PowerFecal Pro DNA Kit (Qiagen, Hilden, Germany) and the saliva samples using QIAamp DNA Microbiome Kit (Qiagen, Hilden, Germany). All protocol procedures were performed according to the manufacturer's instructions. Quantification of extracted DNA was determined fluorometrically on the Qubit 3.0 Fluorometer (Thermo Fisher Scientific, United States) using the QubitTM dsDNA HS Assay Kit. DNA purity was determined via 260/280 and 260/230 ratios measured on the NanoDrop 1000 (Thermo Fisher Scientific, United States). The SMARTer Thruplex DNA‐Seq (Takara Bio) was used for library preparation (Option: 350 bp; Category: low input). Samples were sequenced on NovaSeq6000 (NovaSeq Control Software 1.7.0/RTA v3.4.4) with a 151nt (Read1)‐10nt(Index1)‐10nt(Index2)‐151nt(Read2) setup using “NovaSeqXp” workflow in “S4” mode flow cell. The Bcl to FastQ conversion was performed using bcl2fastq_v2.20.0.422 from the CASAVA software suite. The quality scale used was Sanger /phred33/Illumina 1.8+.

Raw paired‐end metagenomics data were analyzed using Metaphlan3^[^ ^62^ ^]^ to extract each sample's taxonomic profiles. The abundant data were then analyzed using the Wilcoxon rank‐sum test to identify the species different between subjects with no steatosis compared to the other groups. Spearman correlation analysis was used to analyze the associations between selected analytes and the taxonomic abundance data. The correlation between oral and gut metagenomics data (by filtering the species with abundance > 1% in at least five data points) was used. The *SciPy* package was used. All analyses were done using Python 3.

### Untargeted Metabolomics Analysis

Plasma samples were collected for nontargeted metabolite profiling by Metabolon (Durham, NC). The samples were prepared with an automated system (MicroLab STAR, Hamilton Company, Reno, NV). For quality control purposes, a recovery standard was added before the first step of the extraction. To remove protein and dissociated small molecules bound to protein or trapped in the precipitated protein matrix, and to recover chemically diverse metabolites, proteins were precipitated with methanol under vigorous shaking for 2 min (Glen Mills GenoGrinder 2000) and centrifuged. The resulting extract was divided into four fractions: one each for analysis by ultraperformance liquid chromatography‐tandem mass spectroscopy (UPLC‐MS/MS) with positive ion‐mode electrospray ionization, UPLC‐MS/MS with negative ion‐mode electrospray ionization, and gas chromatography‐mass spectrometry; one fraction was reserved as a backup.

### Inflammatory Protein Markers

In the plasma samples, inflammatory protein markers were determined with the Olink Inflammation panel (Olink Bioscience, Uppsala, Sweden). Briefly, each sample was incubated with 92 DNA‐labeled antibody pairs (proximity probes). When an antibody pair bound to its corresponding antigens, the corresponding DNA tails formed an amplicon by proximity extension, which could be quantified by high‐throughput, real‐time polymerase chain reaction (PCR). Probe solution (3 µL) was mixed with 1 µL of sample and incubated overnight at 4 °C. Then 96 µL of extension solution containing extension enzyme and PCR reagents for the preamplification step was added. The extension products were mixed with detection reagents and primers and loaded on the chip for qPCR analysis with the BioMark HD System (Fluidigm Corporation, USA). To minimize inter and intra‐run variation, the data were normalized to both an internal control and an interplate control. Normalized data were expressed in arbitrary units (Normalized Protein eXpression, NPX) on a log2 scale and linearized with the formula 2^NPX^. A high NPX indicated a high protein concentration. The limit of detection, determined for each of the 92 assays, was defined as three standard deviations above the negative control (background).

### Statistical Analysis

Values were expressed as the mean ± standard deviation (SD) (continuous variables) or as *n* (%). For all analyses, metabolites and proteins that were missing in > 50% of patients were removed. Wilcoxon signed‐rank test (two‐sided) was used to compare the difference in gut and oral microbiome between different steatosis groups. Student's *t*‐test (two‐sided) was used to compare the difference in other multiomics between different steatosis groups. The sample sizes of none, mild, moderate, and severe steatosis group are 10, 14, 20, and 12. All the statistical analyses are based on these samples. Missing values were dropped before the analysis. PCA was performed using *scikit‐learn* package. Finally, Spearman correlation analysis was used to analyze the association between selected analytes and other datasets (metabolomics and proteins). The Benjamini–Hochberg method was used to adjust the *p* values. The *SciPy* package was used. All analyses were done using Python 3.7.

### Random Forest Analysis

A random forest classification algorithm was used to find each dataset's key features and each network cluster. The analyses were performed using the *RandomForestClassifier* function from the *scikit‐learn* package. Several trees were calculated before the analysis by selecting the highest accuracy with the lowest number of trees (up to 100 trees). Sample bootstrapping and out‐of‐bag sample options were enabled. AUC‐ROC (receiver operating characteristic) was generated using the same module, by combining the classes (no and mild steatosis, and moderate and severe steatosis)

### Generation of Multiomics Network

Multiomics network was generated based on the Spearman correlations and the significant associations (FDR < 0.05). The analyses were performed with the *SciPy* package in Python 3.7. Centrality analysis and Leiden Clustering (community analysis) on the network were performed using *iGraph* Python and *leidenalg* module. Missing values were omitted in a pairwise manner (using *nan_policy = “omit”* option). Networks were visualized using Cytoscape 3.8.2. All networks presented on this manuscript can be accessed openly in iNetModels (http://inetmodels.com).

### Code Availability

All code used for the analyses is available in https://github.com/sysmedicine/nafldBaseline.

## Conflict of Interest

A.M., J.B. and M.U. are the founders and shareholders of the ScandiBio Therapeutics AB. The other authors declare no conflict of interest.

## Author Contributions

M.Z., M.A., and X.L. contributed equally to this work. Study Design, M.U., J.B., A.M.; Patient Recruitment, M.Z., M.A., B.S., M.G.G.; Investigation, H.Y., O.A., M.A., C.F., W.K., X.L., J.M.S., H.T., C.Z., S.S., J.N.; Data Analyses, O.A., M.A., C.F., W.K., X.L., J.M.S., C.Z., S.S., J.N., A.M.; Interpretation, M.Z., M.U., J.B., A.M.; Drafting the manuscript, M.Z, O.A., A.M.; Revising the manuscript, all authors.

## Supporting information

Supporting Information

Supporting Information

Supporting Information

Supporting Information

Supporting Information

Supporting Information

Supporting Information

Supporting Information

Supporting Information

Supporting Information

Supporting Information

Supporting Information

Supporting Information

Supporting Information

Supporting Information

Supporting Information

Supporting Information

## Data Availability

The data that supports the findings of this study are available in the Supporting Information of this article.
