# Supplementary material for: Multiomics Analysis Reveals the Impact of Microbiota on Host Metabolism in Hepatic Steatosis
Source: Adv Sci (Weinh). 2022 Feb 7;9(11):2104373. doi: 10.1002/advs.202104373 (PMC9008426; doi:10.1002/advs.202104373)
Supplement: Supplementary file 1 — Supporting Information [file ADVS-9-2104373-s013.pdf]

ADVANCED SCIENCE
Open Access

Supporting Information

for *Adv. Sci.*, DOI: 10.1002/advs.202104373

Multi-omics analysis reveals the impact of microbiota on host metabolism in hepatic steatosis

Mujdat Zeybel,[1,2,3,] Muhammad Arif,[4,] Xiangyu Li,[4,] Ozlem Altay,[4] Hong Yang,[4] Mengnan Shi,[4] Murat Akyildiz,[1] Burcin Saglam,[1] Mehmet Gokhan Gonenli,[1] Buket Yigit,[1] Burge Ulukan,[1] Dilek Ural,[5] Saeed Shoaie,[4,7] Hasan Turkez,[8] Jens Nielsen,[9] Cheng Zhang,[4,6] Mathias Uhlén,[4] Jan Borén,[10,] Adil Mardinoglu[4,7,]**

The associations between metagenomics data and clinical parameters

In gut microbiome, our analysis showed that the abundance of species belongs to Bacteroides (*Sanguibacteroides justesenii*) in the gut was negatively correlated with all HS, AST, ALT, GGT and uric acid levels (P<0.05, Figure 3A, Dataset S5). In an extended manner, we observed that the reduced abundance of individual species in Firmicutes (*Firmicutes bacterium* CAG 95, *Firmicutes bacterium* CAG 110, Firmicutes bacterium CAG 238, *Lactobacillus ruminis*, *Phascolarctobacterium* sp CAG 266), Lentisphaerae (*Victivallis vadensis* and Proteobacteria (*Bilophila wadsworthia*), as well as the increased abundance of species, belongs to Proteobacteria (*Haemophilus* sp HMSC71H05) in the gut were significantly correlated with the HS (P<0.05, Figure 3A, Dataset S5). We also found that the abundance of species belongs to Actinobacteria (*Bifidobacterium longum*), Firmicutes (*Ruminococcus obeum* CAG 39 and *Holdemanella biformis*) and Proteobacteria (*Escherichia coli*) was significantly negatively correlated with ALT, AST and GGT levels, however, Firmicutes (*Coprococcus comes*) was significantly negatively correlated only with ALT and AST levels (P<0.05, Figure 3A, Dataset S5).

In oral microbiome, AST and ALT levels were negatively correlated with the abundances of *Bacteroides plebeius* and *Capnocytophaga gingivalis;* HS and GGT were negatively correlated with the abundances of *Capnocytophaga leadbetteri, Streptococcus* sp HPH0090 and *Prevotella marshii*; HS and AST were positively correlated with the abundances of *Oscillibacte*r sp 57 20 and *Bacteroides fragilis*. Moreover, HS positively correlated with the abundances of *Alistipes shahii, Escherichia coli, Neisseria macacae, Actinomyces* sp oral axon 414 as well as negatively correlated with the abundances of *Porphyromonas endodontalis, Streptococcus sp F0442, Prevotella marshii, Treponema sp OMZ 838, Prevotella sp F0091* and *Treponema denticola* (P<0.05, Figure 3B, Dataset S5).

The gut microbiota plays a significant role in uric acid metabolism. We showed that the abundances of *Ruminococcus bromii, Slackia isoflavoniconvertens, Dorea longicatena, Firmicutes* bacterium CAG 95, *Firmicutes* bacterium CAG 110, *Bilophila wadsworthia, Victivallis vadensis, Roseburia* sp CAG 182 and *Phascolarctobacterium* sp CAG 266 in the gut microbiome and the abundances of *Capnocytophaga leadbetteri, Capnocytophaga granulosa, Streptococcus* sp HPH0090 and *Treponema denticola* in the oral microbiome were significantly negatively correlated with both uric acid levels and HS (P<0.05, Figure 3A & Figure 3B, Dataset S5). We also found that the abundances of *Bacteroides* sp CAG 144, *Alloprevotella tannerae, Prevotella jejuni, Streptococcus cristatus* and *Veillonella rogosae* in the gut microbiome and the abundances of *Centipeda periodontii, Prevotella* sp oral taxon 820, *Actinomyces meyeri* and *Desulfobulbus oralis* in the oral microbiome were significantly positively correlated with the uric acid levels (P<0.05, Figure 3A & Figure 3B, Dataset S5). We observed a negative correlation between uric acid levels and the abundances of species belongs to Actinobacteria (*Bifidobacterium angulatum, Bifidobacterium longum* and *Bifidobacterium bifidum*), Bacteroides (*Butyricimonas virosa*) and Firmicutes (*Mitsuokella multacida, Oscillibacter* sp CAG 241, *Firmicutes bacterium* CAG 83, *Megasphaera elsdenii, Blautia obeum, Eisenbergiella tayi* and *Eubacterium* sp CAG 251) in the gut microbiome

(P<0.05, Figure 3A, Dataset S5), and the abundances of species belongs to Bacteroides (*Capnocytophaga sputigena*) in the oral microbiome (P<0.05, Figure 3B, Dataset S5)

The link between the oral and gut microbiome

To study the transitions and interactions between the oral and gut microbiome, we performed correlation analysis between the abundance of species and observed significant correlations between them (P<0.05, Figure 3C, Dataset S6). We found that the abundance of the *Oscillibacter* sp CAG 241, which was significantly reduced in severe steatosis vs no steatosis and negatively associated with uric acid levels in the gut was significantly positively correlated with the abundance of *Prevotella histicola* and *Aggregatibacter segnis* in the oral microbiome. We also found that the abundance of the *Bacteroides uniformis*, significantly increased in moderate steatosis vs no steatosis, in the gut was significantly positively correlated with the abundance of *Rothia mucilaginosa* in the oral microbiome. Similarly, we found that the abundance of the *Roseburia inulinivorans* significantly increased in moderate steatosis vs no steatosis in the gut was significantly positively correlated with the abundance of *Actinomyces odontolyticus* and *Actinomyces sp* HMSC035G02 and significantly negatively correlated with the abundance of *Porphyromonas somerae* and *Neisseria flavescens* in the oral microbiome.

On the other hand, we found that the abundance of *Campylobacter concisus*, significantly negatively associated with HS in the oral microbiome was significantly positively correlated with the abundance of *Alistipes putredinis* and *Collinsella aerofaciens* in the gut microbiome (P<0.05, Figure 3C, Dataset S6). Moreover, we found that that the abundance of *Veillonella atypica*, significantly reduced in mild steatosis vs no steatosis in the oral microbiome was significantly negatively correlated with the abundance of *Parasutterella excrementihominis* in the gut microbiome. Of note, we found that abundances of *Parasutterella excrementihominis* in the gut microbiome were mostly affected by alterations in the abundance of different species in the oral microbiome (P<0.05, Figure 3C, Dataset S6).

The influence of the microbiome on the plasma metabolome

In the gut microbiome, we observed that the abundance *Bacteroides uniformis,* significantly increased in subjects with moderate steatosis and *Oscillibacter* sp CAG 241 that was significantly reduced in subjects with severe steatosis is significantly correlated with the plasma metabolites involved in amino acid metabolism.

In the oral microbiome, the plasma level of phenol glucuronide (tyrosine metabolism) was positively correlated with the abundances of *Prevotella* sp oral taxon 306, *Porphyromonas gingivalis* and *Prevotella intermedia* but negatively correlated with the abundances of *Rothia dentocariosa* and *Streptococcus sanguinis* (Figure S3, Dataset S13). We also observed that the abundance of *Campylobacter concisus*, significantly negatively correlated with HS and *Veillonella atypica*, significantly reduced in subjects with mild steatosis vs no steatosis was significantly correlated with the glutamate and phenol sulfate, respectively. These species' abundances were also associated with the plasma level of metabolites involved in amino acid metabolism, carnitine metabolism, and lipid metabolism.

The plasma level isovalerylcarnitine, associated with BCAA metabolism, was positively correlated with the abundances of *Alloprevotella tannerae*, *Prevotella nigrescens* and *Prevotella oulorum* but negatively correlated with the abundances of *Prevotella histicola* and *Streptococcus infantis*. The plasma level of tiglyl carnitine, associated with BCAA metabolism, was positively correlated with the abundances of *Alloprevotella tannerae* and *Prevotella shahii* but negatively correlated with the abundances of *Streptococcus parasanguinis, Megasphaera micronuciformis, Veillonella atypica, Veillonella dispar* and *Neisseria sicca*.

The plasma level of N6,N6,N6-trimethyllysine (lysine metabolism) was positively correlated with the abundances of *Porphyromonas gingivalis* and *Porphyromonas somerae* but negatively correlated with the abundances of *Veillonella atypica*, *Prevotella histicola, Prevotella salivae* and *Neisseria* sp oral taxon 014. The plasma level of 2,3-dihydroxy-5-methylthio-4-pentenoate (methionine, cysteine, and taurine metabolism) was positively correlated with the abundances of *Prevotella nigrescens* but negatively correlated with the abundances of *Actinomyces odontolyticus*, *Actinomyces* sp HMSC035G02, *Prevotella intermedia* and *Campylobacter concisus*. The plasma level of glutamate (glutamate metabolism) was positively correlated with the abundances of *Prevotella copri* and *Neisseria mucosa* but negatively correlated with the abundances of *Prevotella intermedia, Fusobacterium periodonticum* and *Campylobacter concisus*; urate (purine metabolism) was positively correlated with the abundances of *Prevotella oulorum*, *Prevotella* sp oral taxon 306, *Tannerella* sp oral taxon HOT 286 but negatively correlated with the abundances of P*orphyromonas endodontalis* and *Prevotella intermedia*. All correlations between individual species in the oral microbiome and plasma metabolites presented in Figure S3 and Dataset S13.

The influence of the microbiome on the plasma proteome

In the group with the moderate steatosis, we found that CSF1 was positively correlated with the abundance of *Parasutterella excrementihominis*; TWEAK was positively correlated with the abundance of *Oscillibacter* sp CAG 241; CCL23 was negatively correlated with the abundances of *Roseburia intestinalis*, *Eubacterium eligens*, *Barnesiella intestinihominis* and *Roseburia faecis* (Figure S4, Dataset S14). Additionally, associations of significant proteins in the group with severe steatosis with gut microbiome revealed a negative correlation between HGF plasma level and the abundances of *Butyrivibrio crossotus* and *Roseburia intestinalis*; a negative correlation between NT-3 plasma level and the abundances of *Prevotella* sp CAG 279; a positive correlation between OPG plasma level and the abundances of *Dialister* sp CAG 357 and *Coprococcus eutactus*; a positive correlation between CCL3 plasma level and the abundances of Oscillibacter sp 57 20, *Dialister* sp CAG 357 and *Coprococcus eutactus*; a positive correlation of CCL4 plasma level and the abundances of *Roseburia inulinivorans* and *Coprococcus eutactus,* but a negative correlation with the abundances of *Roseburia intestinalis*; a positive correlation of CCL20 plasma level and the abundances of *Coprococcus eutactus* (Figure S4, Dataset S14).

In the oral microbiome, we found species within *Neisseria* genus (*N. mucosa*, *Neisseria sp oral taxon 014, N. elongate, N. subflava, N. sicca*), *Rothia* genus (*R. aeria, R. dentocariosa,*

R. mucilaginosa) and *Veillonella* genus (*V. parvula, V. atypica*) were positively associated with the numerous inflammatory proteins (Figure S5, Dataset S14). However, there was a negative correlation between the abundance of species belonging to the *Porphyromonas* genus (namely *P. somerae*, *P. endodontalis*, *P. gingivalis*) and the *Prevotella* genus (namely *P. pallens, P. oulorum, P. shahii, P. intermedia*) with the inflammation-related proteins (Figure S5, Dataset S14). Interestingly, the abundances of the *Neisseria flavescens, Haemophilus parainfluenzae* and *Campylobacter concisus* were also negatively correlated with inflammation-related proteins (Figure S5, Dataset S14). Besides, FGF-21 plasma level was negatively correlated with the abundances of *Streptococcus mitis* and *Tannerella* sp oral taxon HOT 286; CDCP1 plasma level was positively correlated with the abundances of *Neisseria mucosa* and negatively correlated with the abundances of *Tannerella* sp oral taxon HOT 286; IL-6 plasma level was negatively correlated with the abundances of *Porphyromonas endodontalis* and CSF-1 plasma level was negatively correlated with the abundances of *Alloprevotella tannerae* (Figure S5, Dataset S14). Other significantly correlated species with plasma inflammation-related proteins are presented in Figure S5, Dataset S14.

SUPPLEMENTARY FIGURE LEGENDS

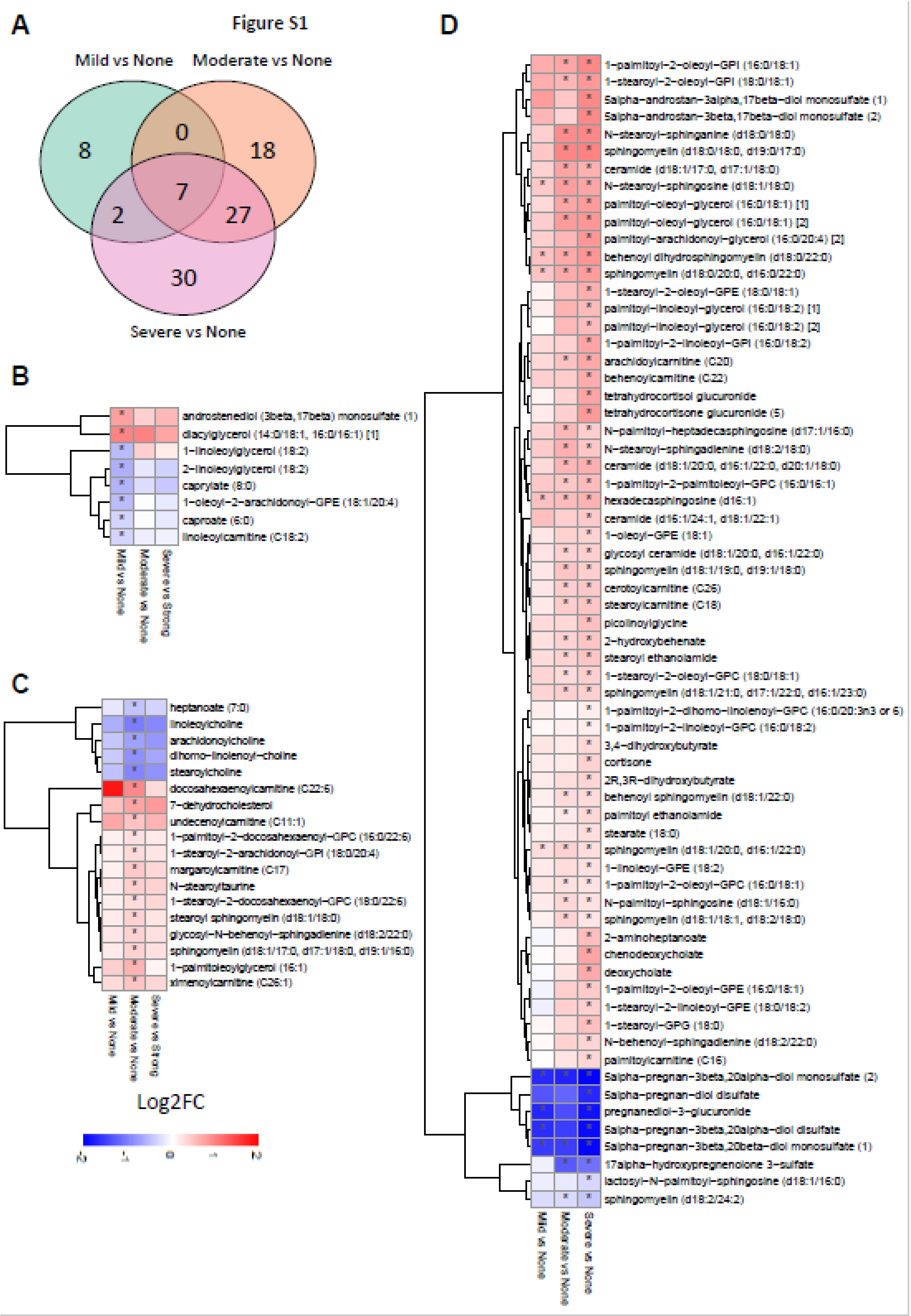

Figure S1 (A) Venn diagram shows significantly altered lipid metabolites in all comparisons. Heatmap shows Log2FC based alterations of metabolites that are exclusively different in the subjects with (B) mild steatosis and (C) moderate steatosis compared to the subjects with no steatosis. (D) All significantly altered lipid metabolites in the subjects with severe steatosis compared to the subjects with no

steatosis. Asterisks indicate statistical significance based on t-test. $P<0.05$. Log2FC: log2(fold change).

Figure S2

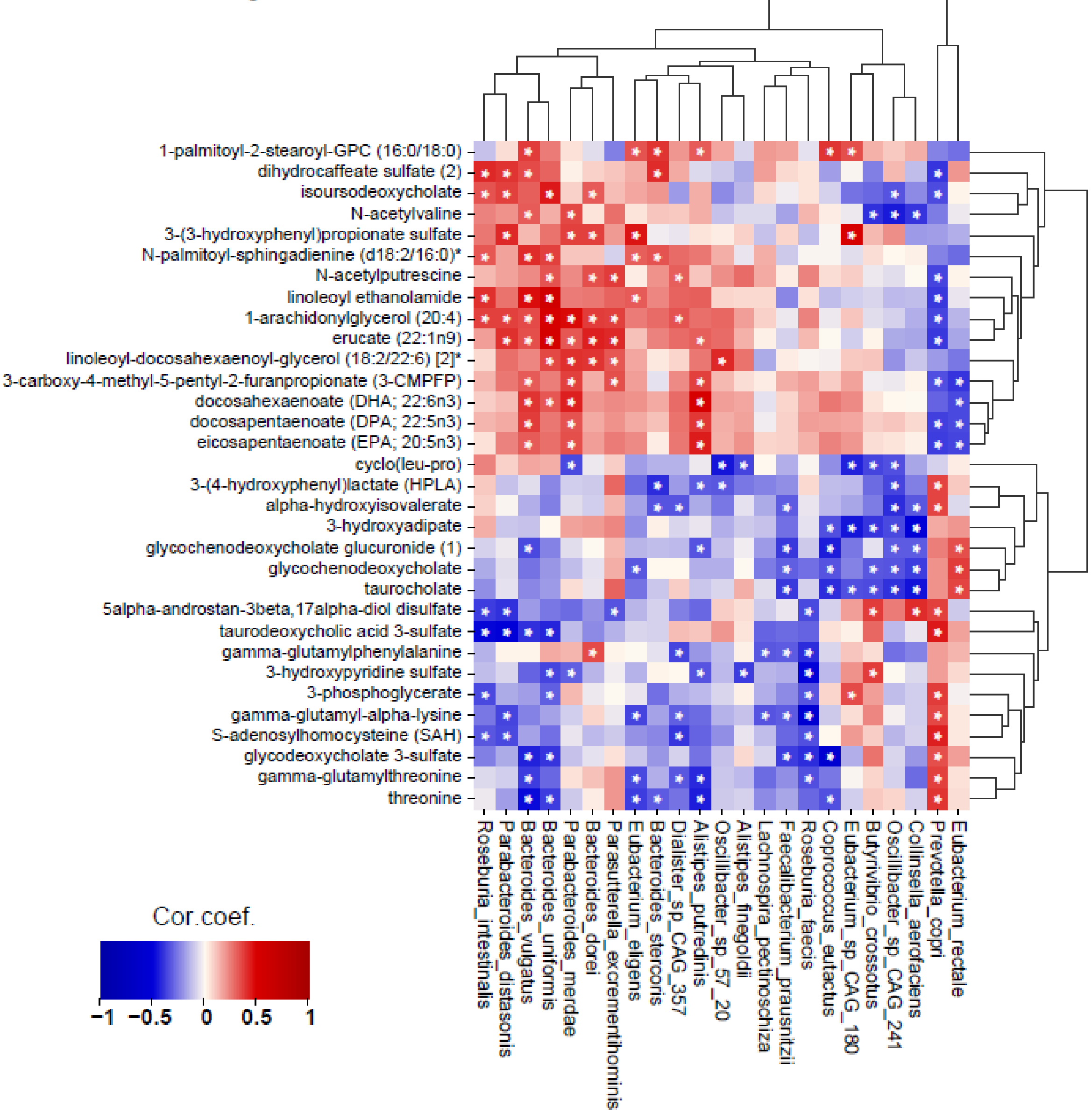

Figure S2 Heatmap is showing the Spearman correlation score (paired) between metabolites and gut microbiota species (abundance > 1%). Asterisks denotes significant correlations (P < 0.05). Only metabolites that were significantly correlated with 5 or more species are shown in the heatmap

Figure S3

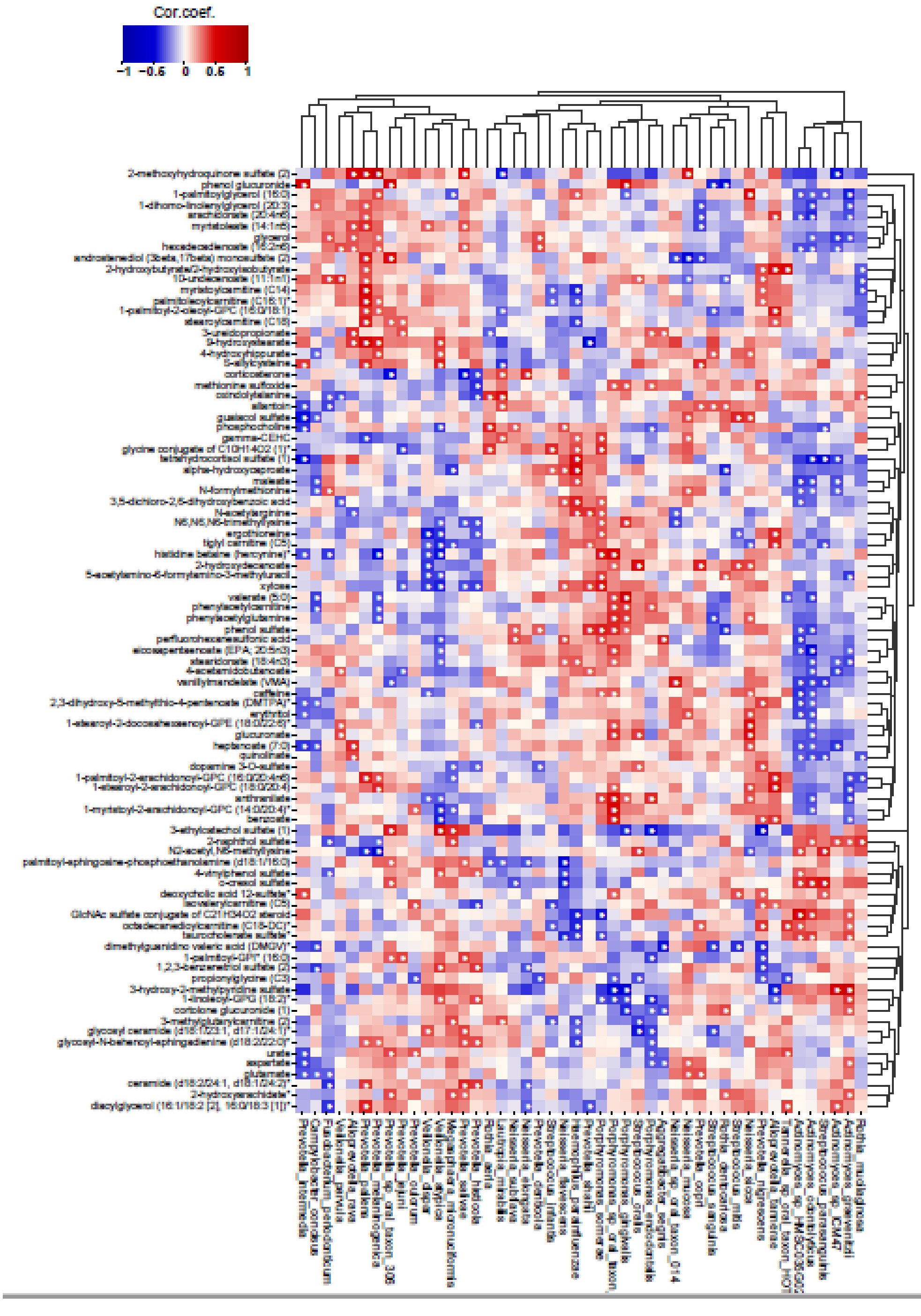

Figure S3 Heatmap is showing the Spearman correlation score (paired) between metabolites and oral microbiota species (abundance > 1%). Asterisks denotes significant correlations (P < 0.05). Only metabolites that were significantly correlated with 5 or more species are shown in the heatmap

Figure S4

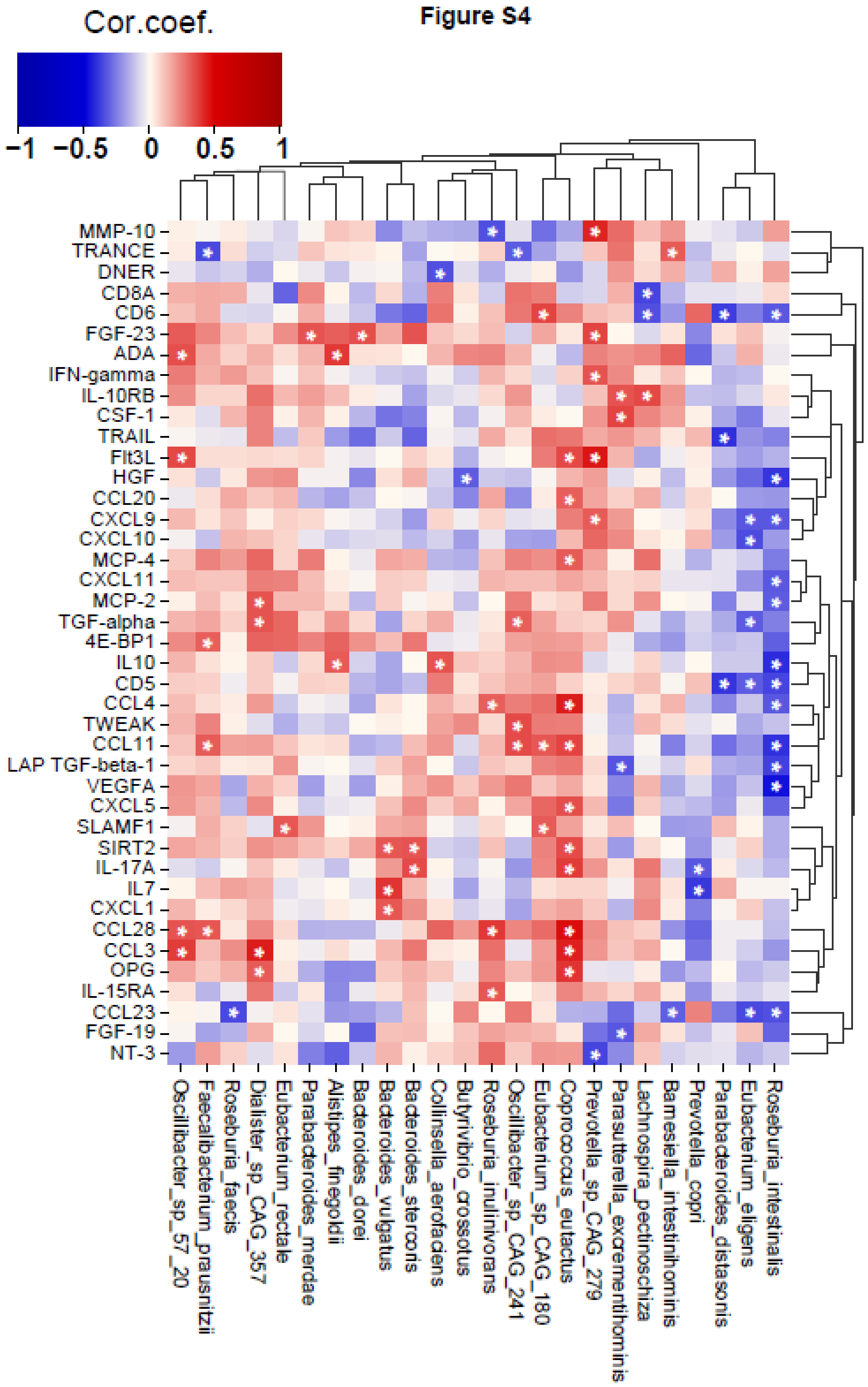

Figure S4 Heatmap is showing the Spearman correlation score (paired) between proteins and gut microbiota species (abundance > 1%). Asterisks denotes significant correlations (P < 0.05).

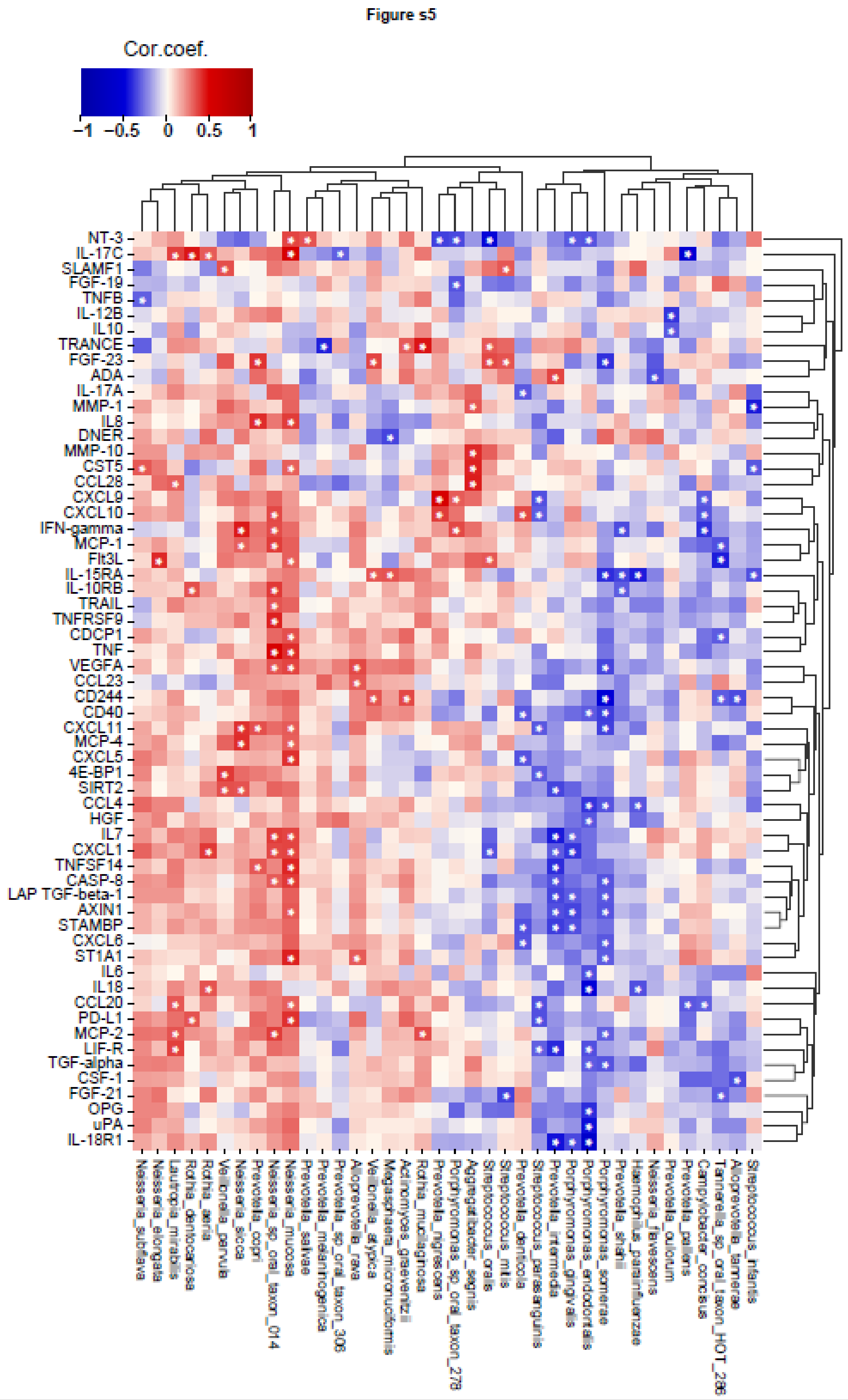

Figure S5 Heatmap is showing the Spearman correlation score (paired) between proteins and oral microbiota species (abundance > 1%). Asterisks denotes significant correlations (P < 0.05).

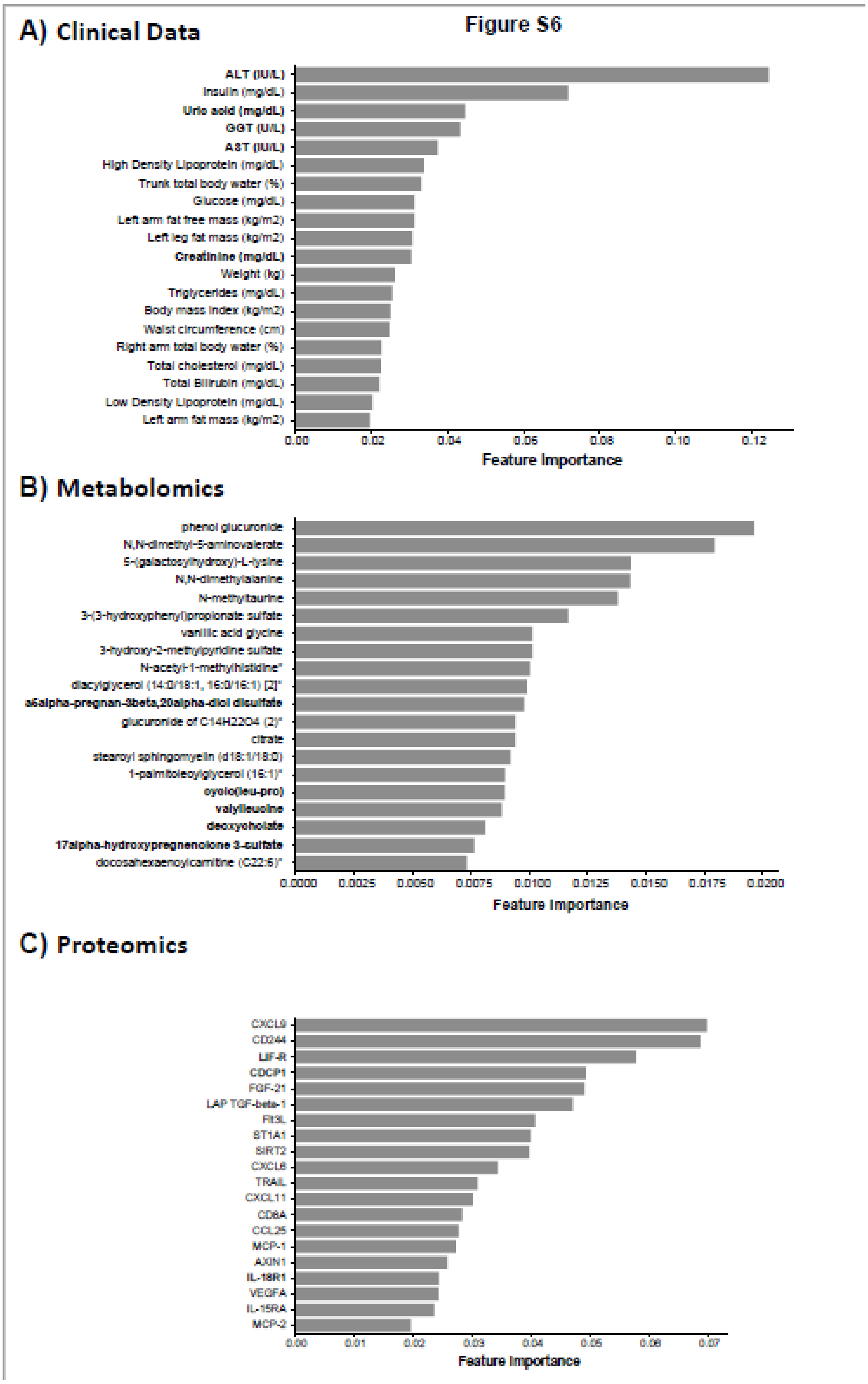

Figure S6 Top 20 features from (A) clinical, (B) metabolomics, and (C) proteomics data identified by random forest classification. Analytes altered significantly ($P < 0.05$) between severe vs no steatosis comparison are marked in bold.

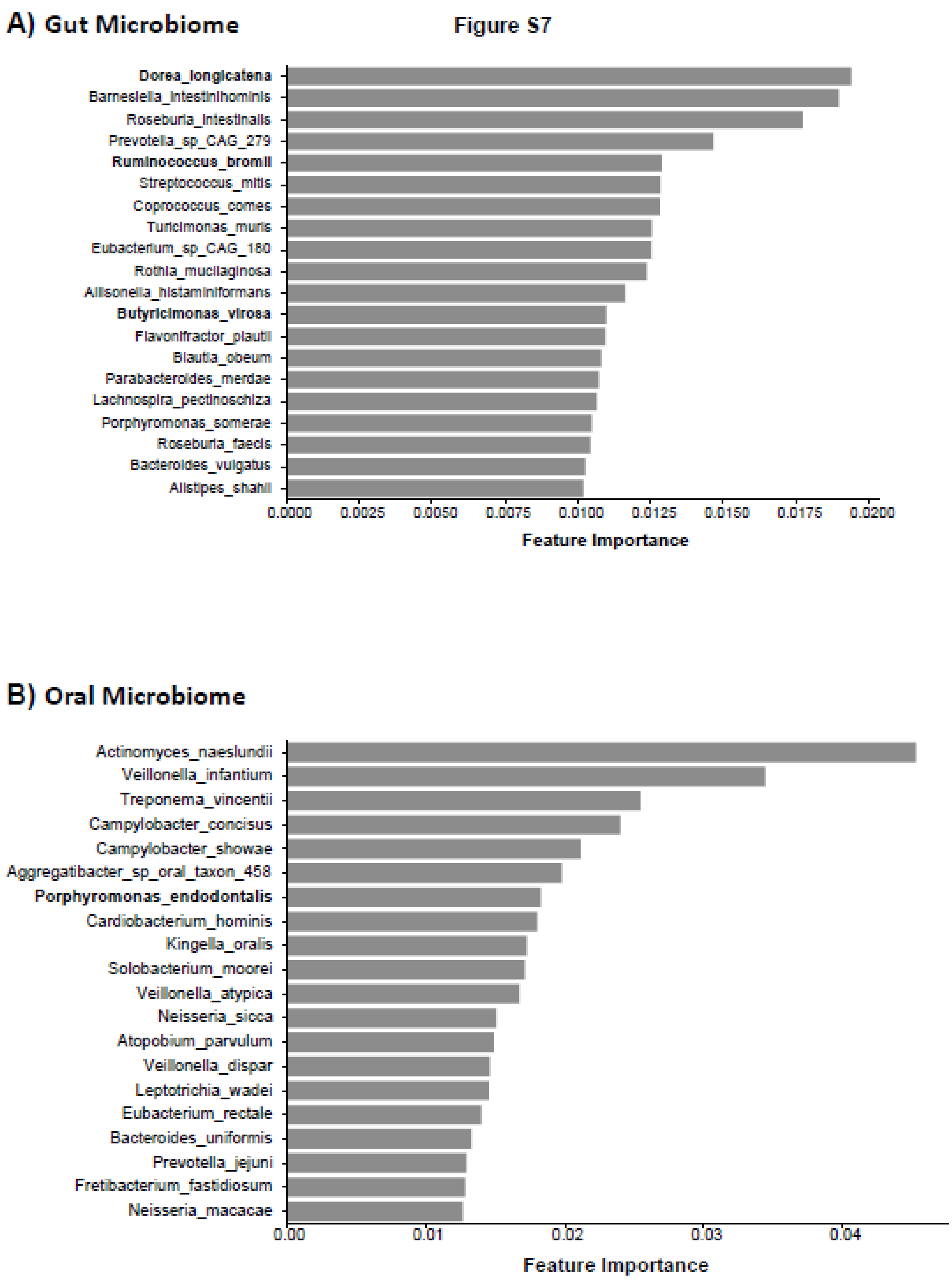

Figure S7 Top 20 features from (A) Gut and (B) Oral microbiome data identified by random forest classification. Analytes altered significantly ($P < 0.05$) between severe vs no steatosis comparison are marked in bold.

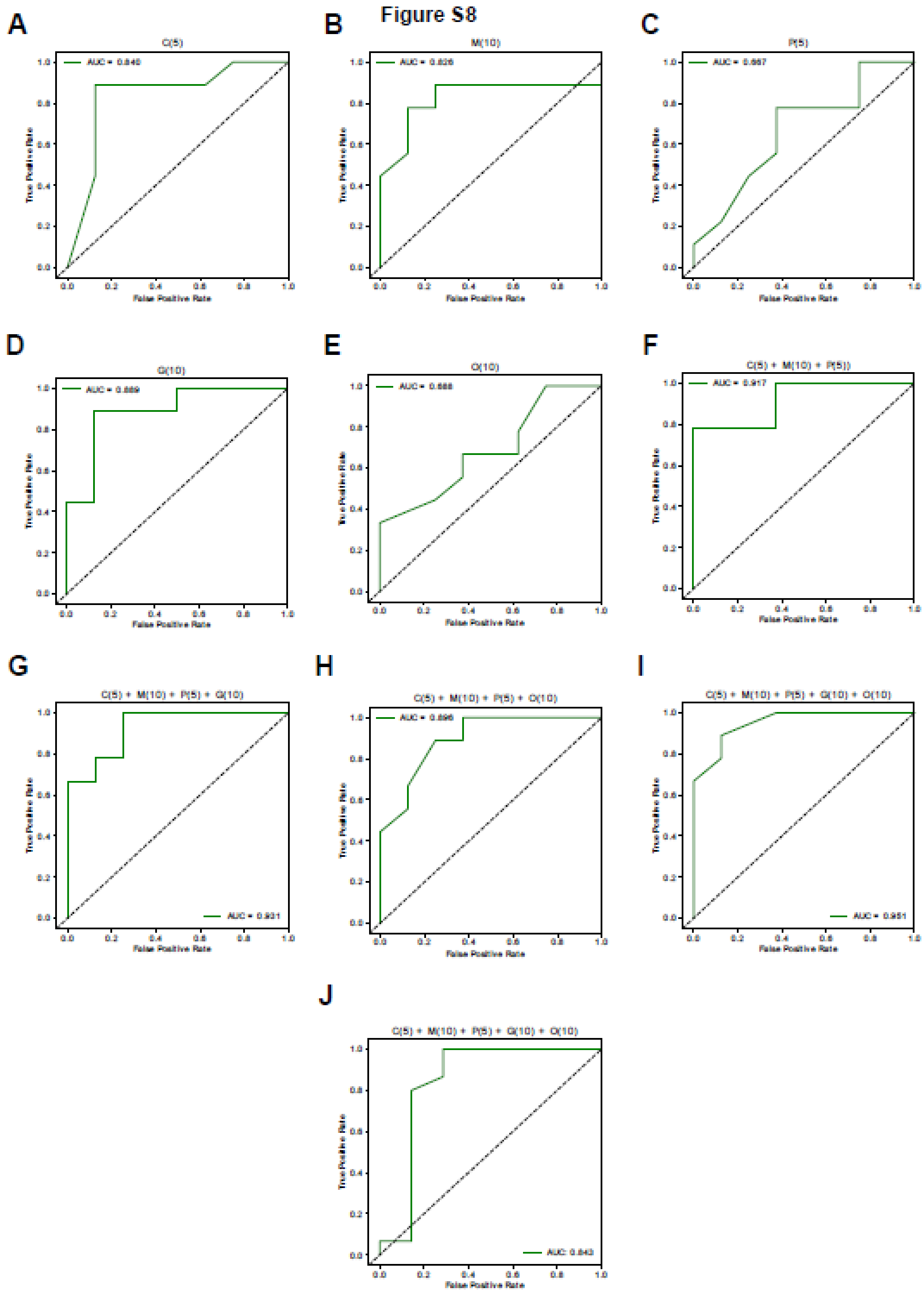

Figure S8 (A) – (I) AUC-ROC curves for HS prediction based on single/multi-omics data based on the data from 56 subjects. (J) AUC-ROC curve for validation of the final model predicts HS based on the data from 22 subjects.

SUPPLEMENTARY DATASETS

Dataset S1 Clinical and physical variables (A-C) are presented for the 56 subjects, recruited in the finding cohort. D) The mean values for these variables and the differences in the clinical and physical variables between subjects with mild, moderate, and severe hepatic steatosis are compared to those with no hepatic steatosis.

Dataset S2 Clinical and physical variables (A-C) and the mean values (D) of the variables are presented for the 22 subjects, recruited in the validation cohort. Multi-omics data, including oral (E) and gut (F) metagenomics, metabolomics (G) and proteomics (H) data generated for the 22 subjects. (I) The difference of clinical characteristics between the overall 56 subjects and the 22 subjects.

Dataset S3 Metagenomics Raw Data for each of 56 subject recruited in the study.

Dataset S4 Differences in the abundance of the species in the gut and oral microbiome between the subjects with mild, moderate and severe hepatic steatosis compared to those with no hepatic steatosis.

Dataset S5 Associations between the abundance of the species in the gut and oral microbiome and the level of significantly altered clinical variables are presented.

Dataset S6 Association between the abundances of species in the gut and oral microbiome is presented.

Dataset S7 Untargeted metabolomics data for each of 56 subject recruited in the study.

Dataset S8 Differences in plasma level of metabolites between the subjects with mild, moderate and severe hepatic steatosis compared to the subjects with no hepatic steatosis. Only metabolites detected in >50% of samples were analysed.

Dataset S9 Associations between the plasma level all metabolites and the level of significantly altered clinical and physical variables are presented.

Dataset S10 The Olink multiplex inflammation panel used to detect the dynamic range of 72 proteins in the subjects' plasma samples.

Dataset S11 Differences in the plasma level of inflammation-related proteins between the subjects with mild, moderate and severe hepatic steatosis compared to the subjects with no hepatic steatosis. Only proteins detected in >50% of samples were analysed.

Dataset S12 Associations between the plasma level of all inflammation-related proteins and the level of significantly altered clinical and physical variables are presented.

Dataset S13 Associations between the plasma level all metabolites and the abundance of the species in the gut and oral microbiome are presented.

Dataset S14 Associations between the plasma level all proteins and the abundance of the gut and oral microbiome species are presented.

Dataset S15 Highly ranked metabolites, proteins, species and clinical features based on Random Forest analysis.

Dataset S16 Multi-Omics Network Data, including edges and nodes information, are presented. The network is shown in the iNetModels (http://inetmodels.com).
